# Chemical Constituents from the Whole Plant of *Cuscuta reflexa*

**DOI:** 10.1007/s13659-020-00265-x

**Published:** 2020-09-21

**Authors:** Tin Thu Thu Aung, Meng-Yuan Xia, Pyae Phyo Hein, Rong Tang, Dong-Dong Zhang, Jun Yang, Xue-Fei Yang, Dong-Bao Hu, Yue-Hu Wang

**Affiliations:** 1 Key Laboratory of Economic Plants and Biotechnology and the Yunnan Key Laboratory for Wild Plant Resources, Kunming Institute of Botany, Chinese Academy of Sciences, Kunming, 650201 People’s Republic of China; 2 School of Chemical Biology and Environment, Yuxi Normal University, Yuxi, 653100 People’s Republic of China; 3 University of Chinese Academy of Sciences, Beijing, 100049 People’s Republic of China; 4 Southeast Asia Biodiversity Research Institute, Chinese Academy of Sciences, Yezin, Nay Pyi Taw 05282 Myanmar

**Keywords:** *Cuscuta reflexa*, Porcine pancreatic lipase, Platelet aggregation, 2*H*-pyran-2-one glucosides, Steroidal glucosides

## Abstract

**Electronic supplementary material:**

The online version of this article (10.1007/s13659-020-00265-x) contains supplementary material, which is available to authorized users.

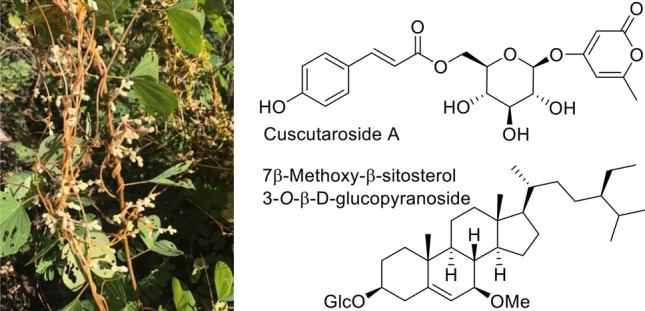

## Introduction

*Cuscuta reflexa* Roxb. (Convolvulaceae), a twining parasitic plant, is distributed in China, Afghanistan, India, Indonesia, Malaysia, Myanmar, Nepal, Pakistan, Sri Lanka, and Thailand [1]. In Nujiang Prefecture, Yunnan Province, China, the Lisu people call it mu-gua-zhua and use its whole plant or seeds to treat soreness and weakness of the waist and knees, erectile dysfunction, spermatorrhea, diabetes, dizziness, hypopsia, and threatened abortion [2]. Named shwe-new or shwe-nwe-pin (Hsay) in Myanmar, the whole plant of *C. reflexa* is used to treat inflammation, irregularities of the blood, and other diseases [3]. It is also medically used in India, Nepal, Bangladesh, and Pakistan [4]. Based on published studies, the major chemical constituents of *C. reflexa* include flavonoids, coumarins, phenylpropanoids, triterpenoids, and cardiac glycosides [4–7].

A previous study showed that extracts of *C. reflexa* possess an antiobesity effect [8], but no further studies clarified the active compounds responsible for the activity. Pancreatic lipase inhibition is one of the most widely studied mechanisms for antiobesity treatment [9]. Purified human lipase (HPL) and porcine pancreatic lipase (PPL) show similar specific activities [10]. Recently, the extract of *C. reflexa* was found to have in vitro thrombolytic activity [11]. This finding may somewhat explain its traditional use in the treatment of irregularities of the blood. However, no active constituents were reported in this research. In the current study, we report the structural elucidation of three new compounds from the whole plant of *C. reflexa* and the results of a bioassay for the inhibitory activities against PPL and rabbit platelet aggregation.

## Results and Discussion

### Structural Elucidation

**Fig. 1:**
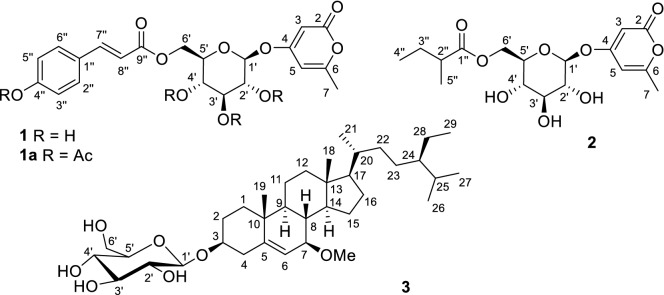
Chemical structures of new compounds (**1**–**3**) and the acetylated derivative of **1**

Through chromatographic techniques, three new compounds (**1**–**3**, Fig. 1) and 12 known compounds (**4**–**15**, Supplementary Material, Fig. S1) were obtained from the EtOH extract of *C. reflexa*.

Based on ^13^C NMR data (Table 1) and the HRESIMS ion peak at *m/z* 457.1106 [M + Na]^+^ (calcd for C_21_H_22_NaO_10_, 457.1111), the molecular formula of **1** was deduced to be C_21_H_22_O_10_. The ^1^H and ^13^C NMR data (Table 1) indicated the presence of a *p*-coumaroyl moiety [*δ*_H_ 7.60 (1H, d, *J* = 15.9 Hz), 7.48 (2H, br d, *J* = 8.7 Hz), 6.79 (2H, br d, *J* = 8.7 Hz), and 6.36 (1H, d, *J* = 15.9 Hz); *δ*_C_ 169.0], one methyl group [*δ*_H_ 2.20 (3H, s); *δ*_C_ 19.8], and one *β*-glucopyranosyl group [*δ*_H_ 5.05 (d, *J* = 7.4 Hz)]. By comparing its NMR data with those of 4-(*β*-d-glucopyranosyloxy)-6-methyl-2*H*-pyran-2-one (**5**) [12], it was implied that compound **1** might be a *p*-coumaroyl derivative of 4-glucopyranosyloxy-6-methyl-2*H*-pyran-2-one. Based on the 2D NMR spectra of **1**, especially the HMBC correlations from H-1ʹ to C-4 and H_2_-6ʹ to C-9ʺ, the 4-glucopyranosyloxy-6-methyl-2*H*-pyran-2-one moiety was confirmed, and the *p*-coumaroyl group was located at 6ʹ-OH through an ester bond. An acetylated derivative (**1a**) was obtained using pyridine and acetic anhydride. We tried to obtain a crystal of **1a**, but unsuccessful. After acidic hydrolysis of **1**, d-glucose was obtained. Thus, the chemical structure of **1** was determined as shown in Fig. 1 and named cuscutaroside A.

**Table 1:** ^1^H and ^13^C NMR data of **1** and **2** in methanol-*d*_4_ (*δ* in ppm, *J* in Hz)

No.	**1**		**2**	
	*δ*_H_ (500 MHz)	*δ*_C_ (125 MHz)	*δ*_H_ (600 MHz)	*δ*_C_ (150 MHz)
2		167.4, C		167.2, C
3	5.73 (d, 2.0)	91.9, CH	5.66 (d, 2.1)	91.6, CH
4		171.3, C		171.3, C
5	6.08 (m)	101.5, CH	6.10 (m)	101.5, CH
6		164.8, C		164.8, C
7	2.20 (s)	19.8, CH_3_	2.25 (s)	19.7, CH_3_
1′	5.05 (d, 7.4)	100.7, CH	5.05 (d, 7.5)	100.6, CH
2′	3.48 (m)	74.3, CH	3.43 (dd, 9.3, 7.5)	74.3, CH
3′	3.49 (m)	77.7, CH	3.47 (dd, 9.3, 8.7)	77.5, CH
4′	3.39 (dd, 9.4, 8.9)	71.5, CH	3.33 (dd, 9.7, 8.7)	71.5, CH
5′	3.78 (m)	76.0, CH	3.73 (m)	75.8, CH
6′	4.55 (dd, 12.0, 2.0) 4.24 (dd, 12.0, 7.3)	64.6, CH_2_	4.43 (dd, 11.9, 2.1) 4.17 (dd, 11.9, 7.1)	64.5, CH_2_
1″		127.1, C		178.2, C
2″	7.48 (br d, 8.7)	131.4, CH	2.43 (m)	42.3, CH
3″	6.79 (br d, 8.7)	116.9, CH	1.65 (m) 1.47 (m)	27.9, CH_2_
4″		161.5, C	0.97 (t, 7.3)	11.9, CH_3_
5″	6.79 (br d, 8.7)	116.9, CH	1.13 (d, 7.0)	17.0, CH_3_
6″	7.48 (br d, 8.7)	131.4, CH		
7″	7.60 (d, 15.9)	147.0, CH		
8″	6.36 (d, 15.9)	114.7, CH		
9″		169.0, C		

**Fig. 2:**
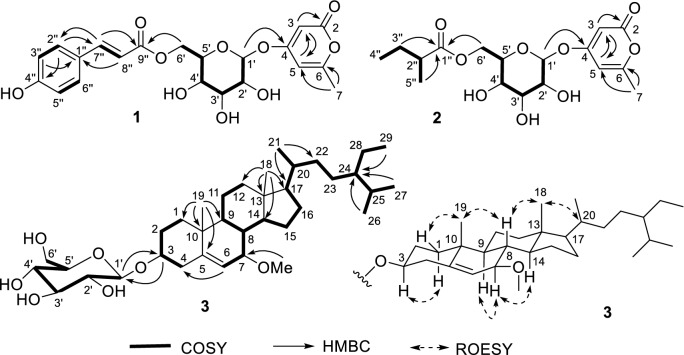
Key 2D NMR correlations of compounds **1**–**3**

The molecular formula of cuscutaroside B (**2**) was determined to be C_17_H_24_O_9_ based on the ^13^C NMR data (Table 1) and the HRESIMS ion peak at *m/z* 395.1313 [M + Na]^+^ (calcd for C_17_H_24_NaO_9_, 395.1318). The ^1^H and ^13^C NMR data of **2** (Table 1) indicated the presence of one 6-methyl-2*H*-pyran-2-one moiety (*δ*_C_ 171.3, 167.2, 164.8, 101.5, and 91.6) [12], one *β*-glucopyranosyl group [*δ*_H_ 5.05 (d, *J* = 7.5 Hz)], and one 2-methylbutyryl group (*δ*_C_ 178.2, 42.3, 27.9, 17.0, and 11.9) [13]. Compound **2** was deduced as a 2-methylbutyryl derivative of 4-(*β*-glucopyranosyloxy)-6-methyl-2*H*-pyran-2-one by the COSY and HMBC correlations (Fig. 2). Based on the HMBC correlations from H_2_-6ʹ to C-1ʺ, the 2-methylbutyryl group was located at 6ʹ-OH. Thus, the structure of **2** (cuscutaroside B) was elucidated to be 4-[*β*-6-*O*-(2-methylbutyryl)-glucopyranosyloxy]-6-methyl-2*H*-pyran-2-one. We were unable to calculate the ECD spectrum of **2**. The absolute configuration of C-2ʺ remains unknown.

Based on ^13^C NMR (Table 2) and HRESIMS data, compound **3** was determined to have the molecular formula C_36_H_62_O_7_. The ^1^H and ^13^C NMR data (Table 2) of **3** indicated the presence of one *β*-glucopyranosyl group [*δ*_H_ 4.39 (d, *J* = 7.8 Hz)], one methoxy group [*δ*_H_ 3.28 (3H, s); *δ*_C_ 54.9], six methyl groups [*δ*_H_ 1.07 (3H, s), 0.96 (3H, d, *J* = 6.5 Hz), 0.84 (3H, d, *J* = 6.9 Hz), 0.88 (3H, d, *J* = 7.2 Hz), 0.87 (3H, t, *J* = 7.0 Hz), and 0.72 (3H, s)], and one trisubstituted double bond [*δ*_H_ 5.48 (t, *J* = 1.7 Hz); *δ*_C_ 145.7 and 122.5]. Comparison of its NMR data with those of 7-oxo-*β*-sitosterol 3-*O*-*β*-d-glucopyranoside (**4**, Table 2) and 7*β*-hydroxysitosterol-3-*O*-*β*-d-glucopyranoside indicated that compound **3** might be a 7-oxygenated derivative of daucosterol [14–16], which was confirmed by the COSY and HMBC correlations (Fig. 2). According to the HMBC correlations from the OMe group to C-7, H-3 to C-1ʹ, and H-1ʹ to C-3, the methoxy group and the glucopyranosyloxy group were located at C-7 and C-3, respectively. The relative configuration of compound **3** was partially determined by ROESY correlations (Fig. 2) and coupling constants (Table 2). ROESY correlations of H-1*β*/H_3_-19, H_3_-19/H-8, H-8/H_3_-18, and H_3_-18/H-20 were observed, indicating that these protons are cofacial, and thus, H-1α and H-17 are α-oriented. H-7 and H-8 should be in a *trans* axial relationship because of a large coupling constant for H-7/H-8 (*J* = 8.5 Hz), and thus, H-7 is α-oriented. The ROESY correlations of H-1α/H-3, H-9/H-7, and H-7/H-14 indicated that H-3, H-9, and H-14 are also α-oriented. The configurations of C-20 and C-24 on the side chain could not be determined by the ROESY correlations. Because the NMR data of the side chain were highly consistent with those in the literature [14–17], the configurations of C-20 and C-24 were suggested to be the same as those in daucosterol. Finally, compound **3** was elucidated to be 7*β*-methoxy-*β*-sitosterol 3-*O*-*β*-glucopyranoside.

**Table 2:** ^1^H (800 MHz) and ^13^C (200 MHz) NMR data of **3** and **4** in methanol-*d*_4_ (*δ* in ppm, *J* in Hz)

No.	**3**		**4**	
	*δ*_H_	*δ*_C_	*δ*_H_	*δ*_C_
1*β* 1α	1.88 (m) 1.05 (m)	38.2, CH_2_	2.00 (m) 1.25 (m)	39.9, CH_2_
2	1.96 (m) 1.63 (m)	30.7, CH_2_	2.07 (m) 1.73 (m)	30.4, CH_2_
3	3.62 (m)	79.4, CH	3.76 (m)	78.5, CH
4α 4*β*	2.50 (ddd, 13.3, 4.7, 2.1) 2.30 (br t, 13.3)	39.4, CH_2_	2.68 (m) 2.47 (ddd, 13.5, 11.8, 2.2)	39.6, CH_2_
5		145.7, C		168.8, C
6	5.48 (t, 1.7)	122.5, CH	5.69 (d, 1.2)	126.5, CH
7	3.44 (dt, 8.5, 2.0)	83.4, CH		204.7, C
8	1.52 (m)	38.3, CH	2.32 (dd, 12.7, 10.9)	46.6, CH
9	1.05 (m)	49.9, CH	1.52 (m)	51.5, CH
10		37.8, C		37.4, C
11	1.52 (m) 1.40 (m)	22.3, CH_2_	1.64 (m)	22.3, CH_2_
12*β* 12α	2.04 (dt, 12.8, 3.8) 1.16 (m)	41.0, CH_2_	2.08 (m) 1.16 (m)	40.1, CH_2_
13		44.0, C		44.3, C
14	1.16 (m)	57.7, CH	1.32 (m)	51.5, CH
15	1.71 (m) 1.40 (m)	26.8, CH_2_	1.26 (m)	27.4, CH_2_
16	1.88 (m) 1.29 (m)	29.6, CH_2_	1.89 (m) 1.30 (m)	29.6, CH_2_
17	1.11 m	56.9, CH	1.12 (m)	56.1, CH
18	0.72 (s)	12.3, CH_3_	0.73 (s)	12.3, CH_3_
19	1.07 (s)	19.4, CH_3_	1.25 (s)	17.7, CH_3_
20	1.38 (m)	37.4, CH	1.38 (m)	37.5, CH
21	0.96 (d, 6.5)	19.4, CH_3_	0.97 (d, 6.6)	19.5, CH_3_
22	1.38 (m) 1.05 (m)	35.1, CH_2_	1.38 (m) 1.05 (m)	35.1, CH_2_
23	1.21 (m)	27.2, CH_2_	1.22 (m)	27.2, CH_2_
24	0.95 (m)	47.3, CH	0.96 (m)	47.3, CH
25	1.69 (m)	30.4, CH	1.68 (m)	30.4, CH
26	0.84 (d, 6.9)	19.4, CH_3_	0.84 (d, 6.9)	19.4, CH_3_
27	0.88 (d, 7.2)	20.2, CH_3_	0.87 (d, 7.2)	20.2, CH_3_
28	1.33 (m)	24.2, CH_2_	1.32 (m)	24.2, CH_2_
29	0.87 (t, 7.0)	12.3, CH_3_	0.90 (t, 7.1)	12.4, CH_3_
1′	4.39 (d, 7.8)	102.5, CH	4.40 (d, 7.8)	102.7, CH
2′	3.15 (dd, 9.1, 7.8)	75.1, CH	3.15 (dd, 9.0, 7.8)	75.1, CH
3′	3.36 (m)	78.1, CH	3.36 (dd, 9.0, 8.6)	78.1, CH
4′	3.27 (m)	71.7, CH	3.27 (m)	71.7, CH
5′	3.26 (m)	77.9, CH	3.27 (m)	78.0, CH
6′	3.85 (dd, 11.7, 1.5) 3.65 (dd, 11.7, 5.3)	62.8, CH_2_	3.86 (dd, 11.9, 1.7) 3.65 (dd, 11.9, 5.4)	62.8, CH_2_
7-OMe	3.28 (s)	54.9, CH_3_		

The NMR data of 7-oxo-*β*-sitosterol 3-*O*-*β*-d-glucopyranoside (**4**) in pyridine-*d*_5_ and CDCl_3_ have been reported previously [14, 15]. Its NMR data in methanol-*d*_4_ are presented in Table 2. The other known compounds, 4-(*β*-d-glucopyranosyloxy)-6-methyl-2*H*-pyran-2-one (**5**) [12], 4-hydroxyacetophenone (**6**) [18], piceoside (**7**) [19], scrophenoside B (**8**) [20], methyl 4,5-di-*O*-caffeoylquinate (**9**) [21], methyl 3,5-di-*O*-caffeoylquinate (**10**) [21], methyl 3,4-di-*O*-caffeoylquinate (**11**) [21], (6*S*,9*R*)-roseoside (**12**) [22], methyl *trans*-*p*-hydroxycinnamate (**13**) [23], ethyl *trans*-*p*-hydroxycinnamate (**14**) [24], and *N-trans*-feruloyltyramine **(15**) [25], were determined by comparing their NMR data with those in the literature.

### Porcine Pancreatic Lipase and Platelet Aggregation Inhibition Assay

Compounds **1**–**15** were evaluated for their inhibitory activity against PPL. 7*β*-Methoxysitosterol 3-*O*-*β*-glucopyranoside (**3**) showed weak PPL inhibitory activity (IC_50_ = 67.2 ± 1.7 μg/mL) compared with the positive control orlistat (IC_50_ = 0.40 ± 0.02 ng/mL). 7-Oxo-*β*-sitosterol 3-*O*-*β*-d-glucopyranoside (**4**) showed 12 ± 2% inhibition at a concentration of 100 μg/mL. The other tested compounds were inactive, with inhibition values less than 10% at a concentration of 100 μg/mL.

Compounds **1**–**15** and **1a** were also evaluated for their inhibitory activity against rabbit platelet aggregation induced by thrombin (1 U/mL), platelet-activating factor (PAF, 0.4 μg/mL), arachidonate (AA, 0.5 mM), or collagen (4 μg/mL). Cuscutaroside A (**1**), **1a**, and scrophenoside B (**8**) showed weak inhibitory activity against rabbit platelet aggregation induced by collagen with IC_50_ values of 291.4 ± 47.9 μg/mL, 63.8 ± 4.4 μg/mL, and 180.5 ± 6.7 μg/mL, respectively, compared with aspirin (IC_50_ = 33.3 ± 1.3 μg/mL). Compound **1a** also showed inhibitory activity against rabbit platelet aggregation induced by AA with an IC_50_ value of 72.6 ± 10.5 μg/mL compared with aspirin (inhibition 88.1 ± 1.1% at 40 μg/mL). The other tested compounds were inactive (IC_50_ > 300 μg/mL).

## Experimental Section

### General Experimental Procedures

Optical rotations were recorded using a JASCO P-1020 Polarimeter (Jasco Corp., Tokyo, Japan). UV spectra were obtained using a Shimadzu UV-2401 PC spectrophotometer (Shimadzu, Kyoto, Japan). ECD spectra were recorded on a Chirascan CD spectrometer (Applied Photophysics Ltd., Leatherhead, UK). ^1^H and ^13^C NMR spectra were collected on DRX-500, Avance III-600, and Ascend™ 800 MHz spectrometers (Bruker Corp., Karlsruhe, Germany) with TMS as an internal standard. ESIMS and HRESIMS analyses were performed on an API QSTAR Pulsar 1 spectrometer (Applied Biosystems/MDS Sciex, Foster City, CA, USA). EIMS and HREIMS were performed on a Waters AutoSpec Premier p776 spectrometer (Waters, Milford, MA, USA). Silica gel G (80–100 and 300–400 mesh, Qingdao Meigao Chemical Co., Ltd., Qingdao, China), C_18_ silica gel (40–75 μm, Fuji Silysia Chemical Ltd., Aichi, Japan), and Sephadex LH-20 (GE Healthcare Bio-Sciences AB, Uppsala, Sweden) were used for column chromatography, and silica gel GF_254_ (Qingdao Meigao Chemical Co., Ltd.) on precoated plates was used for preparative thin layer chromatography (TLC). TLC spots were visualized under UV light at 254 nm and by dipping them into 5% H_2_SO_4_ in alcohol followed by heating. Semipreparative high-performance liquid chromatography (HPLC) was performed with an Agilent 1200 series pump (Agilent Technologies, Santa Clara, USA) equipped with a diode array detector and an Agilent Zorbax SB-C_18_ column (5.0 μm, *ϕ* 9.4 × 250 mm) and a Welch Ultimate AQ-C_18_ column (5.0 μm, *ϕ* 4.6 × 300 mm).

### Plant Material

The plant material, growing on a *Bauhinia* plant, was collected near Golden Cave (20° 55′ 44.25″ N and 96° 38′ 57.41″ E) in Pindaya Township, Southern Shan State, Myanmar, in December 2016. It was identified as *Cuscuta reflexa* Roxb. by Dr. Jie Cai and Ms. Jun Yang at the Kunming Institute of Botany, Chinese Academy of Sciences. A voucher specimen (no. MMR631) was deposited at the Southeast Asia Biodiversity Research Institute, Chinese Academy of Sciences, Yezin, Nay Pyi Taw, Myanmar and a copy was placed at the Key Laboratory of Economic Plants and Biotechnology, Kunming Institute of Botany, Chinese Academy of Sciences, China.

### Extraction and Isolation

The air-dried powdered whole plant of *Cuscuta reflexa* (3.3 kg) was ultrasonically extracted for 30 min with 70% EtOH at 60 °C. The EtOH extract (519.0 g) was suspended in H_2_O and further partitioned with petroleum ether, EtOAc, and *n*-butanol to the yield petroleum ether-soluble portion (6.4 g, A), the EtOAc-soluble portion (66.8 g, B), and the *n*-butanol soluble portion (276.6 g, C), respectively.

Part B was subjected to column chromatography (silica gel; CH_2_Cl_2_–MeOH, 50:1 → 0:1, v/v) to yield four fractions (B1–B4). Fraction B1 was separated on an RP-C_18_ silica gel column eluted with MeOH–H_2_O (10% → 100%) to yield three major subfractions. The 20% MeOH-eluted portion was purified by Sephadex LH-20 column chromatography (MeOH) and silica gel column chromatography (petroleum ether-EtOAc, 10:1) to yield **6** (8.9 mg). The 40% MeOH-eluted portion was purified by column chromatography (Sephadex LH-20, MeOH), preparative TLC (petroleum ether-acetone, 2:1), and semipreparative HPLC [Agilent Zorbax SB-C_18_ column, MeCN-H_2_O (containing 0.05% TFA), 35:65, 2 mL/min] to yield **13** (5.0 mg, *t*_R_ = 19.388 min) and **14** (2.0 mg, *t*_R_ = 32.159 min).

Fraction B2 was separated on an RP-C_18_ silica gel column eluted with MeOH–H_2_O (10% → 100%) to yield one main subfraction. The 30% MeOH-eluted portion was isolated by Sephadex LH-20 (MeOH) and silica gel column chromatography (CH_2_Cl_2_–MeOH, 30:1) and further purified by semipreparative HPLC [Welch Ultimate AQ-C_18_ column, MeCN-H_2_O (containing 0.05% TFA), 20:80, 1 mL/min] to yield **15** (19.4 mg, *t*_R_ = 36.402 min).

Fraction B3 was separated on an RP-C_18_ silica gel column eluted with MeOH-H_2_O (10% → 100%) to yield two main subfractions (B3-1 and B3-2). B3-1 (40% MeOH-eluted portion) was separated by silica gel column chromatography (CH_2_Cl_2_–MeOH, 100:1 and 50:1) to yield B3-1-1 and B3-1-2. B3-1-1 was separated by Sephadex LH-20 (MeOH) and silica gel column chromatography (CH_2_Cl_2_–MeOH, 3:1) followed by semipreparative HPLC (Agilent Zorbax SB-C_18_ column, MeOH–H_2_O, 40:60, 2 mL/min) to yield **2** (3.0 mg, *t*_R_ = 24.052 min) and **8** (2.1 mg, *t*_R_ = 37.642 min). B3-1-2 was separated by Sephadex LH-20 (MeOH) and silica gel column chromatography (CH_2_Cl_2_–acetone, 3:1 → 1:1) to yield **1** (571.5 mg) and a mixture. The mixture was further purified by semipreparative HPLC [Agilent Zorbax SB-C_18_ column, MeCN–H_2_O (containing 0.05% TFA), 28:72, 2 mL/min] to yield **9** (5.9 mg, *t*_R_ = 13.042 min), **10** (5.8 mg, *t*_R_ = 17.294 min), and **11** (2.6 mg, *t*_R_ = 19.588 min). B3-2 (90% MeOH-eluted portion) was separated by Sephadex LH-20 column chromatography (MeOH) and semipreparative HPLC (Welch Ultimate AQ-C_18_ column, MeOH–H_2_O, 87:13, 1 mL/min) to yield **4** (3.5 mg, *t*_R_ = 42.732 min) and **3** (2.0 mg, *t*_R_ = 64.797 min).

Fraction B4 was separated on an RP-C_18_ silica gel column eluted with MeOH–H_2_O (10% → 100%) to yield three main fractions. The 10% MeOH-eluted portion was purified by silica gel column chromatography (CH_2_Cl_2_–MeOH, 30:1 and 20:1) and Sephadex LH-20 column chromatography (MeOH) to yield **5** (1.1 g) recrystallized from MeOH. The 15% MeOH-eluted portion was isolated by silica gel column chromatography (CH_2_Cl_2_–MeOH, 30:1 and 20:1) and Sephadex LH-20 column chromatography (MeOH) and further purified by semipreparative HPLC (Welch Ultimate AQ-C_18_ column, MeOH–H_2_O, 15:85, 1 mL/min) to yield **12** (6.4 mg, *t*_R_ = 36.095 min). The 20% MeOH-eluted portion was isolated by Sephadex LH-20 column chromatography (MeOH) and semipreparative HPLC (Agilent Zorbax SB-C_18_ column, MeOH–H_2_O, 15:85, 2 mL/min) to yield **7** (1.6 mg, *t*_R_ = 22.265 min).

### Spectroscopic Data of Compounds

#### Cuscutaroside A (1)

White powder; $\left[ \alpha \right]_{{\text{D}}}^{{21}}$ − 46.2 (*c* = 0.19, MeOH); UV (MeOH) *λ*_max_ (log*ε*) 311 (4.42), 227 (4.11), 200 (4.49) nm; ^1^H NMR and ^13^C NMR data see Table 1; ESIMS (negative) *m/z* 433 [M-H]^-^, 867 [2 M-H]^-^; HRESIMS (positive) *m/z* 457.1106 [M + Na]^+^ (calcd for C_21_H_22_NaO_10_, 457.1111).

#### Cuscutaroside B (2)

Colorless solid; $\left[ \alpha \right]_{{\text{D}}}^{{21}}$ − 77.6 (*c* = 0.11, MeOH); UV (MeOH) *λ*_max_ (log*ε*) 285 (3.54), 198 (4.22) nm; ECD (*c* 0.011, MeOH) *λ*_max_ (Δ*ε*) 281 (− 0.91), 199 (− 4.06) nm; ^1^H NMR and ^13^C NMR data see Table 1; ESIMS (positive) *m/z* 395 [M + Na]^+^, 767 [2M + Na]^+^; HRESIMS (positive) *m/z* 395.1313 [M + Na]^+^ (calcd for C_17_H_24_NaO_9_, 395.1318).

#### 7*β*-Methoxy-*β*-sitosterol 3-*O*-*β*-glucopyranoside (3)

Colorless solid; $\left[ \alpha \right]_{{\text{D}}}^{{21}}$ − 25.2 (*c* = 0.11, MeOH); ^1^H NMR and ^13^C NMR data see Table 2; ESIMS (positive) *m/z* 629 [M + Na]^+^; HRESIMS (positive) *m/z* 629.4388 [M + Na]^+^ (calcd for C_36_H_62_NaO_7_, 629.4393).

### Acidic Hydrolysis of Compound 1

Compound **1** (20.0 mg, 0.046 mM) was dissolved in 15 mL of 6% aq. HCl and hydrolyzed under reflux (5 h) at 90 °C. Then, the acidic solution was evaporated in vacuo to dryness and separated by silica gel column chromatography eluted with CHCl_3_–MeOH (10:1) to yield d-glucopyranose (5.5 mg, 0.031 mM, 67% yield), which was identified based on its ^1^H NMR spectrum and optical rotation value of [α]_D_^20^  + 40.0 (*c* 0.09, H_2_O) [26].

### Acetylation of Compound 1

Compound **1** (20.6 mg, 0.048 mmol) was dissolved in 500 µL of pyridine and 500 µL of acetic anhydride and stirred for 24 h at room temperature. Then, water (2 mL) was added to the reaction mixture, followed by extraction with ethyl acetate (4 mL). The upper layer was dried under reduced pressure and purified by silica gel column chromatography (petroleum ether-EtOAc, 10:1) to yield **1a** (20.4 mg, 0.034 mmol, 71% yield). White solid; ^1^H NMR (500 MHz, CDCl_3_) *δ* 7.68 (1H, d, *J* = 16.0 Hz, H-7ʺ), 7.61 (2H, br d, *J* = 8.6 Hz, H-2ʺ and H-6ʺ), 7.12 (2H, br d, *J* = 8.6 Hz, H-3ʺ and H-5ʺ), 6.46 (1H, d, *J* = 16.0 Hz, H-8ʺ), 5.80 (1H, br s, H-5), 5.63 (1H, d, *J* = 2.2 Hz, H-3), 5.28 (2H, m), 5.16 (2H, m), 4.37 (1H, dd,* J* = 12.6, 2.3 Hz, H-6ʹa), 4.27 (1H, dd,* J* = 12.6, 6.2 Hz, H-6ʹb), 3.94 (1H, m, H-5ʹ), 2.30 (3H, s), 2.19 (3H, s), 2.06 (3H, s), 2.06 (3H, s), 2.03 (3H, s); ^13^C NMR (125 MHz, CDCl_3_) *δ* 170.2, 169.5, 169.3, 169.2, 168.1, 166.4, 164.4, 163.3, 152.4, 145.2, 132.0, 130.5, 129.7, 122.3, 117.1, 100.1, 96.9, 91.4, 73.0, 72.6, 70.7, 68.2, 62.1, 21.3, 20.7, 20.7, 20.7, 20.0; ESIMS (positive) *m/z* 625 [M + Na]^+^; HRESIMS (positive) *m/z* 625.1528 [M + Na]^+^ (calcd for C_29_H_30_NaO_14_, 625.1533).

### In vitro Porcine Pancreatic Lipase Inhibition Assay

For lipase inhibition tests of each compound, porcine pancreatic lipase was used. *p*-Nitrophenyl butyrate (*p*-NPB) was used as the substrate. First, 5 μL of the lipase solution (40 U/mL) in Tris–HCl buffer (100 mM Tris–HCl, 5 mM CaCl_2_; pH 7.0) was added to a 96-well microtiter plate. Each compound in 1 μL of DMSO and 184 μL of Tris–HCl buffer were added and mixed with the enzyme buffer to start the reaction. After incubation at 37 °C for 15 min, 10 μL of the substrate solution (10 mM *p*-NPB in dimethyl formamide) was added. The enzymatic reaction was carried out for 15 min at 37 °C. The hydrolysis of *p*-NPB to *p*-nitrophenol was monitored at 400 nm using a spectrophotometer [27].

### In Vitro Platelet Aggregation Assay

The inhibitory effects of the compounds against rabbit platelet aggregation induced by thrombin (1 U/mL), PAF (0.4 μg/mL), AA (0.5 mM), or collagen (4 μg/mL) were evaluated according to published methods [28–30].

## Conclusion

Three new and 12 known compounds were isolated from the whole plants of *Cuscuta reflexa* (Convolvulaceae) collected from Myanmar. 7*β*-Methoxy-*β*-sitosterol 3-*O*-*β*-glucopyranoside (**3**) showed weak PPL inhibitory activity (IC_50_ = 67.2 ± 1.7 μg/mL). Cuscutaroside A (**1**), its acetylated derivative (**1a**), and scrophenoside B (**8**) showed weak inhibitory activity against rabbit platelet aggregation induced by collagen (4 μg/mL) with IC_50_ values of 291.4 ± 47.9 μg/mL, 63.8 ± 4.4 μg/mL, and 180.5 ± 6.7 μg/mL, respectively. Compound **1a** also showed inhibitory activity against rabbit platelet aggregation induced by AA (0.5 mM) with an IC_50_ value of 72.6 ± 10.5 μg/mL. These results support previous findings in pharmacological studies related to the traditional uses of the plant.

## Electronic supplementary material

Below is the link to the electronic supplementary material.

Supplementary file1 (PDF 1402 kb)
