# Supplementary material for: Chemical Constituents from the Whole Plant of Cuscuta reflexa
Source: Nat Prod Bioprospect. 2020 Sep 21;10(5):337–44. doi: 10.1007/s13659-020-00265-x (PMC7520498; doi:10.1007/s13659-020-00265-x)

Supplementary Material for

Chemical Constituents from the Whole Plant of *Cuscuta reflexa*

Tin Thu Thu Aung[1,3] · Meng-Yuan Xia[1] · Pyae Phyo Hein[1,3] · Rong Tang[1] · Dong-Dong Zhang[1,4] · Jun Yang[1,4] · Xue-Fei Yang[1,4] · Dong-Bao Hu[2] · Yue-Hu Wang[1,4]

Tin Thu Thu Aung and Meng-Yuan Xia contributed equally to this work.

Electronic supplementary material The online version of this article (doi:) contains supplementary material, which is available to authorized users.

✉ Dong-Bao Hu

lh@yxnu.edu.cn

✉ Yue-Hu Wang

wangyuehu@mail.kib.ac.cn

1 Key Laboratory of Economic Plants and Biotechnology and the Yunnan Key Laboratory for Wild Plant Resources, Kunming Institute of Botany, Chinese Academy of Sciences, Kunming 650201, People's Republic of China

2 School of Chemical Biology and Environment, Yuxi Normal University, Yuxi 653100, People's Republic of China

3 University of Chinese Academy of Sciences, Beijing 100049, People's Republic of China

4 Southeast Asia Biodiversity Research Institute, Chinese Academy of Sciences, Yezin, Nay Pyi Taw 05282, Myanmar

Contents

**Fig. S1.** Chemical structures of known compounds (**4**–**15**) from *Cuscuta reflexa*.
**Fig. S2.** ^{1}H NMR spectrum of **1** (methanol-d_4, 500 MHz).
**Fig. S3.** ^{13}C NMR spectrum of **1** (methanol-d_4, 125 MHz).
**Fig. S4.** HSQC spectrum of **1**.
**Fig. S5.** ^{1}H–^{1}H COSY spectrum of **1**.
**Fig. S6.** HMBC spectrum of **1**.
**Fig. S7.** HRESIMS spectrum of **1.**
**Fig. S8.** ^{1}H NMR spectrum of **2** (methanol-d_4, 600 MHz).
**Fig. S9.** ^{13}C NMR spectrum of **2** (methanol-d_4, 150 MHz).
**Fig. S10.** HSQC spectrum of **2**.
**Fig. S11.** ^{1}H–^{1}H COSY spectrum of **2**.
**Fig. S12.** HMBC spectrum of **2**.
**Fig. S13.** ECD spectrum of **2.**
**Fig. S14.** HRESIMS spectrum of **2.**
**Fig. S15.** ^{1}H NMR spectrum of **3** (methanol-d_4, 800 MHz).
**Fig. S16.** ^{13}C NMR spectrum of **3** (methanol-d_4, 200 MHz).
**Fig. S17.** HSQC spectrum of **3**.
**Fig. S18.** ^{1}H–^{1}H COSY spectrum of **3**.
**Fig. S19.** HMBC spectrum of **3**.
**Fig. S20.** ROESY spectrum of **3**.
**Fig. S21.** HRESIMS spectrum of **3.**

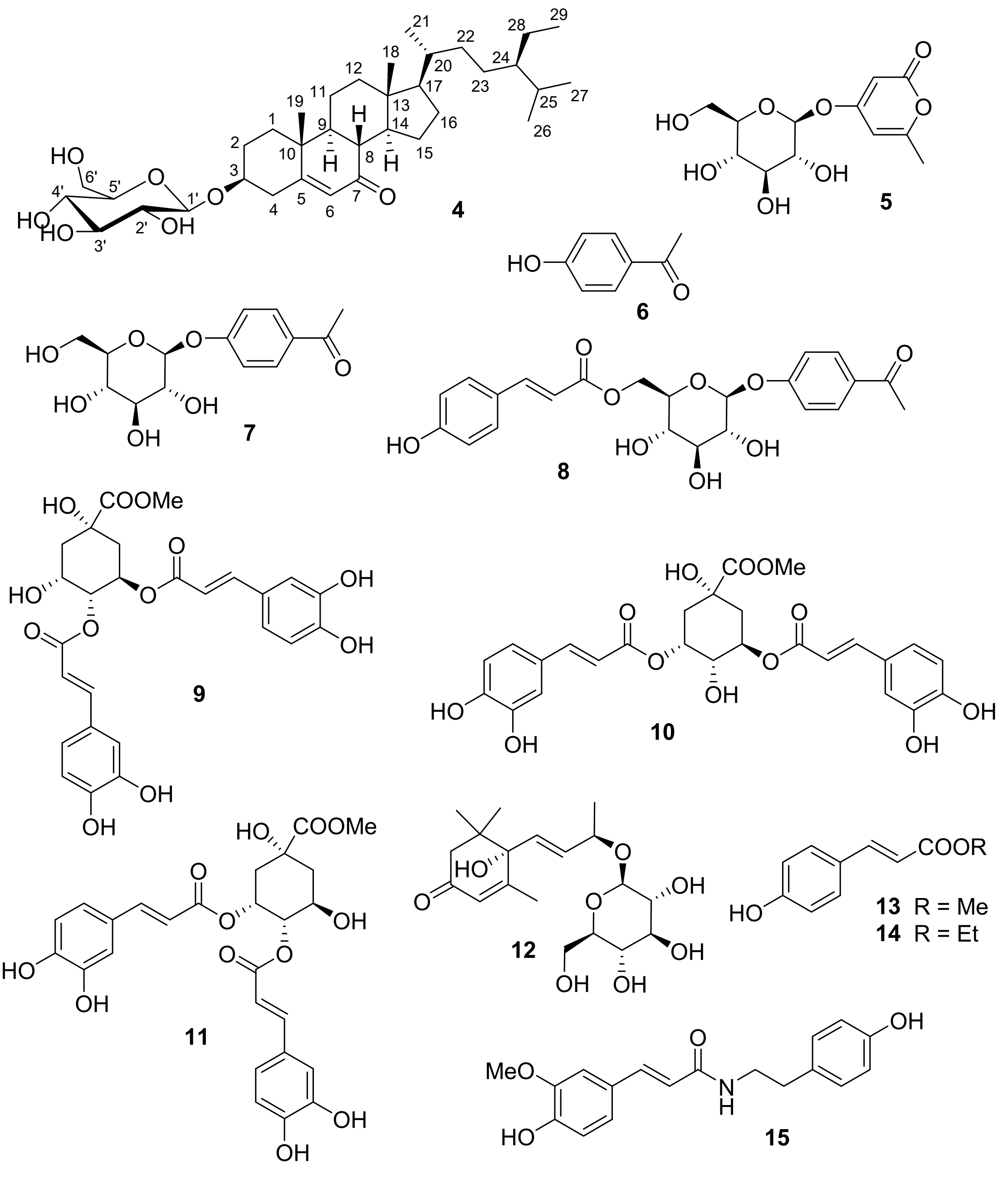

Fig. S1. Chemical structures of known compounds (**4**–**15**) from *Cuscuta reflexa*.

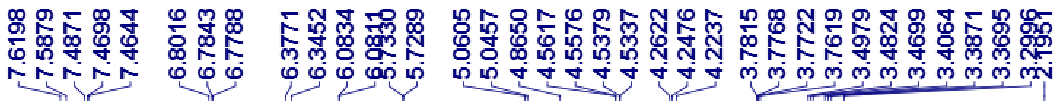

Fig. S2. ^{1}H NMR spectrum of **1** (methanol-d_4, 500 MHz).

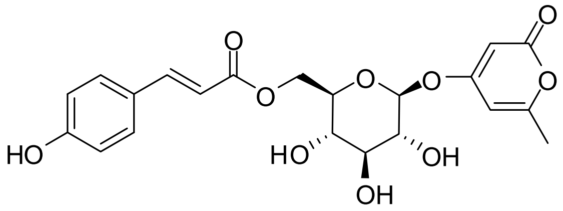

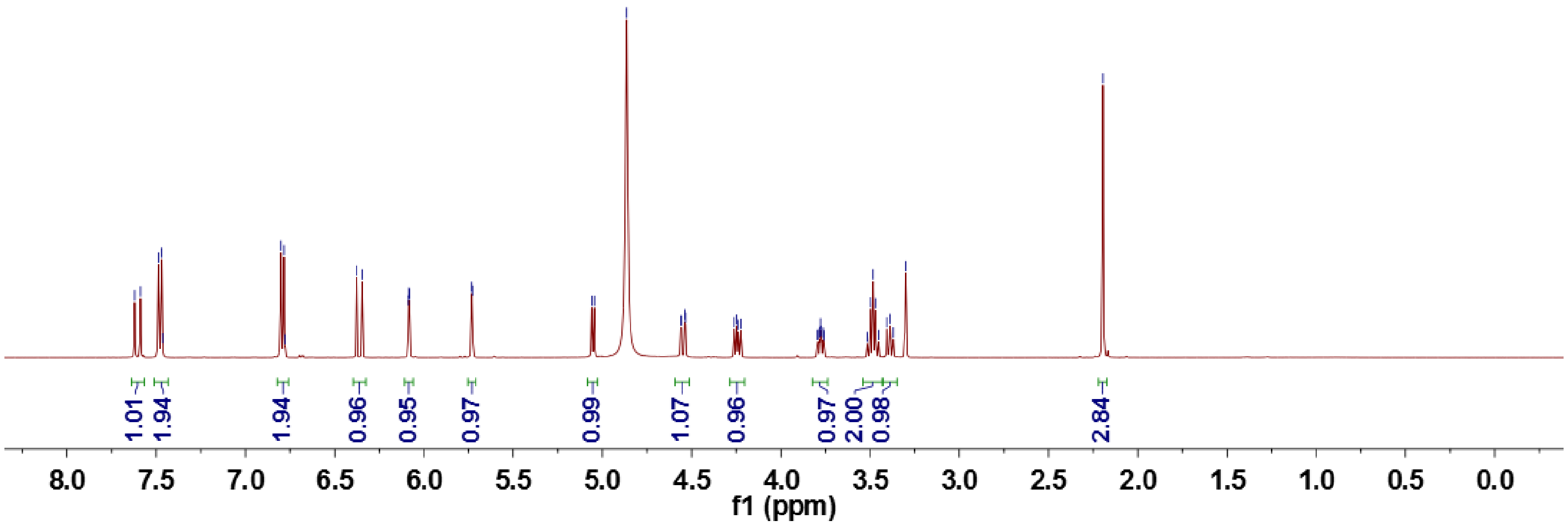

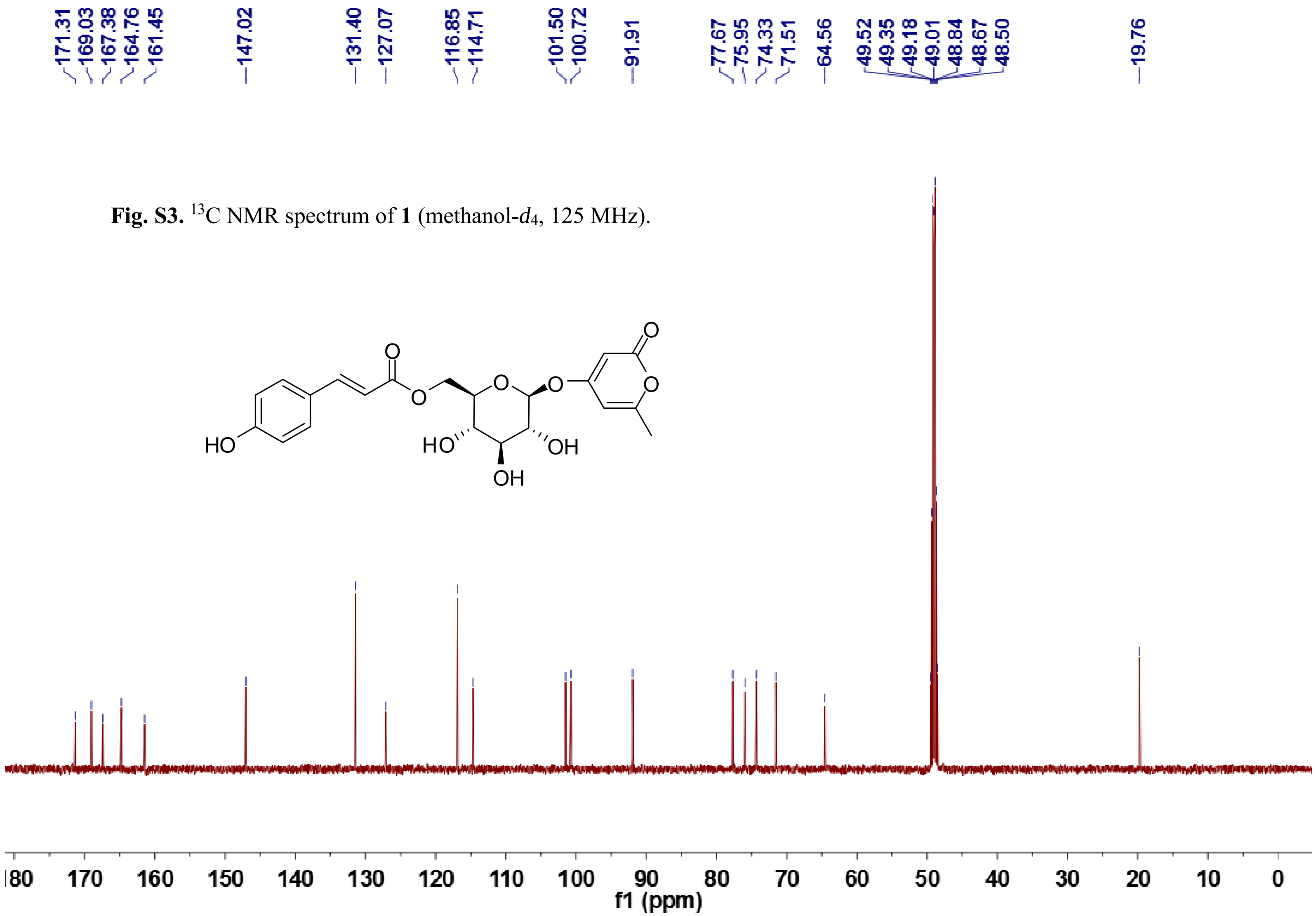

Fig. S3. ^{13}C NMR spectrum of **1** (methanol-d_4, 125 MHz).

Fig. S4. HSQC spectrum of **1**.

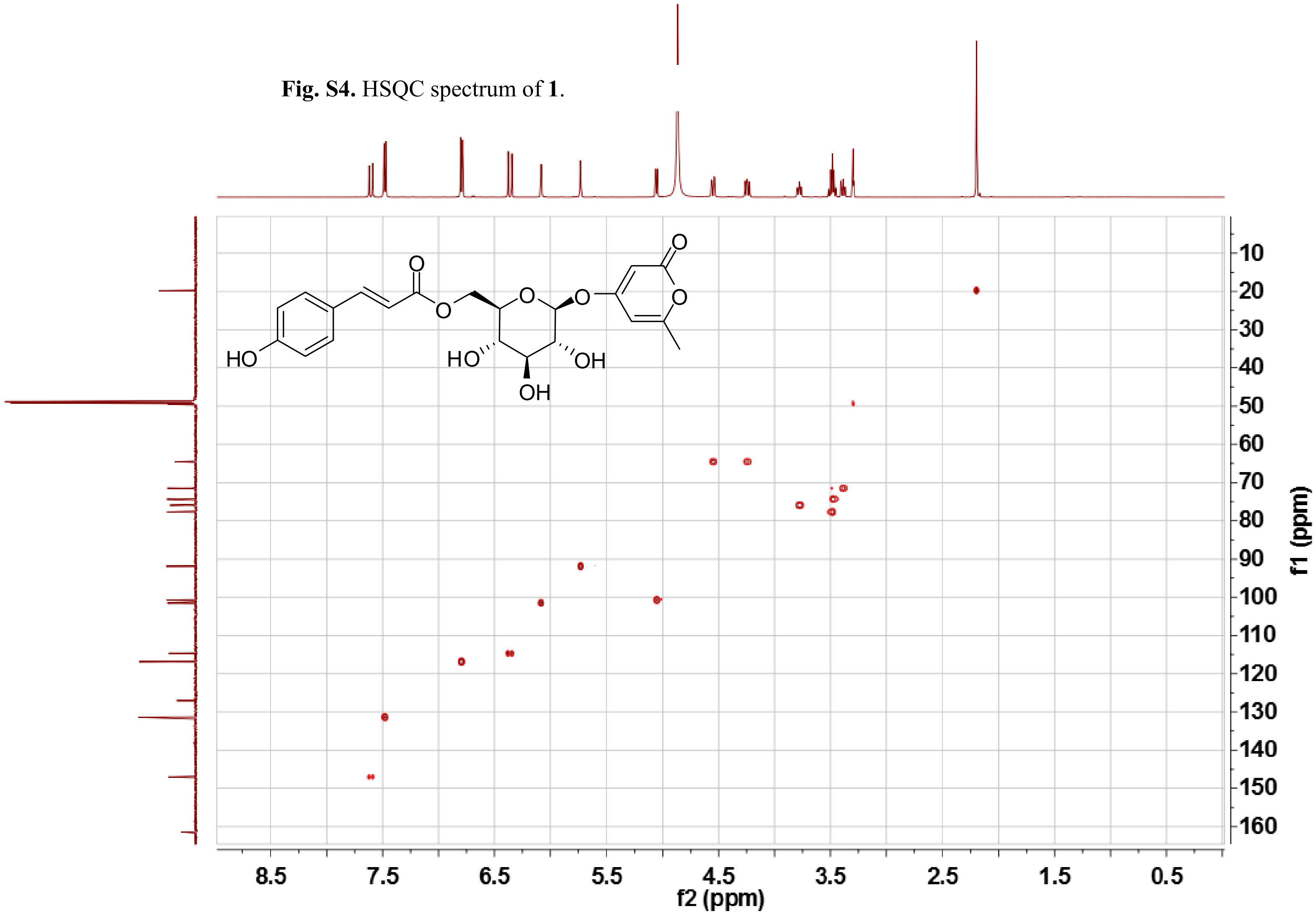

Fig. S5. ^1H–^1H COSY spectrum of **1**.

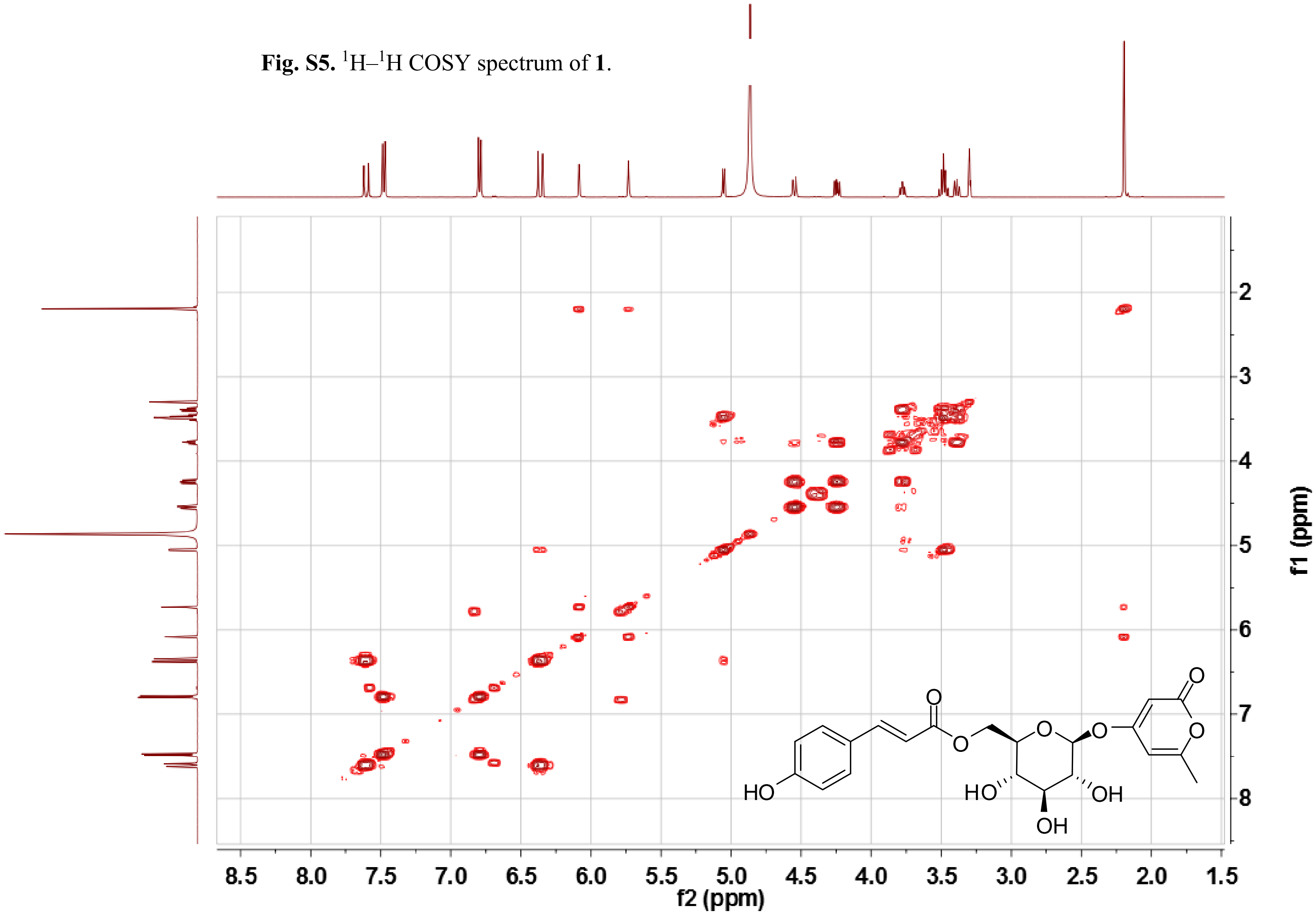

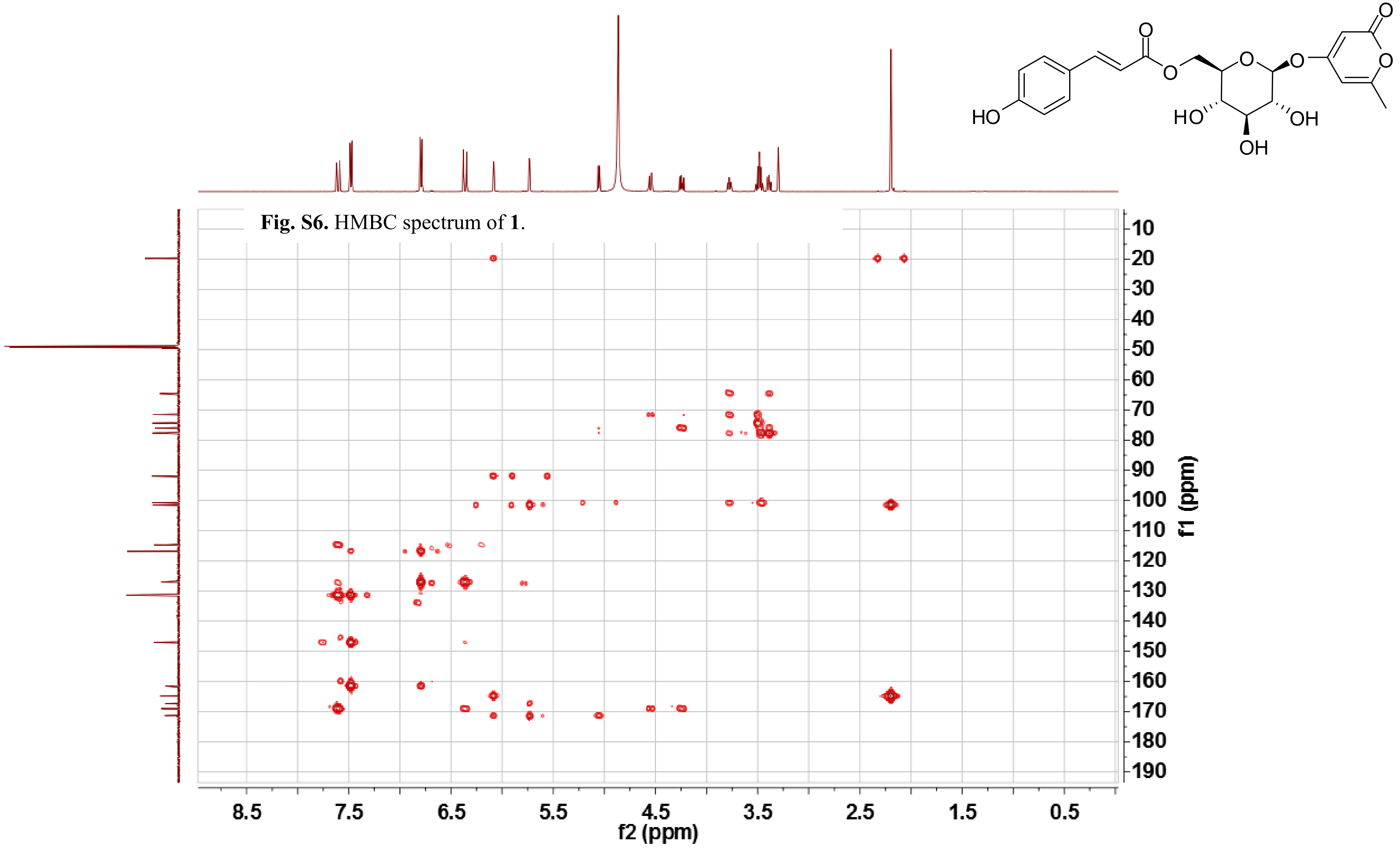

Fig. S6. HMBC spectrum of **1**.

**Sample Type**	Sample	**Position**	
**Instrument Name**	Agilent G6230 TOF MS	**User Name**	KIB
**Acq Method**	ESI.m	**Acquired Time**	3/19/2019 4:52:38 PM
**IRM Calibration Status**	Success	**DA Method**	ESI.m
**Comment**			

**Sample Group**		**Info.**
**Acquisition SW Version**	6200 series TOF/6500 series Q-TOF B.05.01 (B5125.2)	

User Spectra

Fragmentor Voltage	Collision Energy	Ionization Mode
200	0	ESI

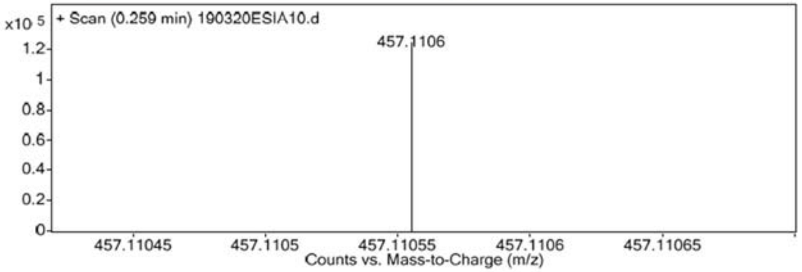

Peak List

m/z	z	Abund	Formula	Ion
102.1282		14751.69		
121.0509	1	127648.15		
457.1106	1	124158.27	C21 H22 Na O10	M+
458.1136	1	27317.52	C21 H22 Na O10	M+
891.231	1	79564.98		
892.2339	1	35277.21		
893.2379	1	11384.33		
922.0098	1	67299.03		
923.0118	1	12252.47		
1325.3503	1	11334.58		

Formula Calculator Element Limits

Element	Min	Max
C	0	200
H	0	400
O	0	15
Na	1	1

Formula Calculator Results

Formula	CalculatedMass	Mz	Diff.(mDa)	Diff. (ppm)	DBE
C21 H22 Na O10	457.1111	457.1106	0.5	1.0	10.5

--- End Of Report ---

Fig. S7. HRESIMS spectrum of **1.**

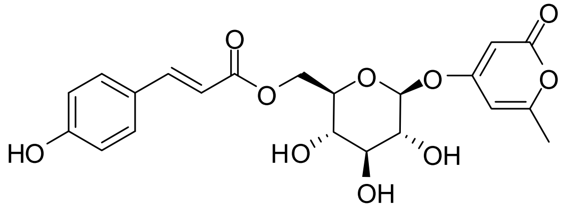

Fig. S8. ^{1}H NMR spectrum of **2** (methanol-d_4, 600 MHz).

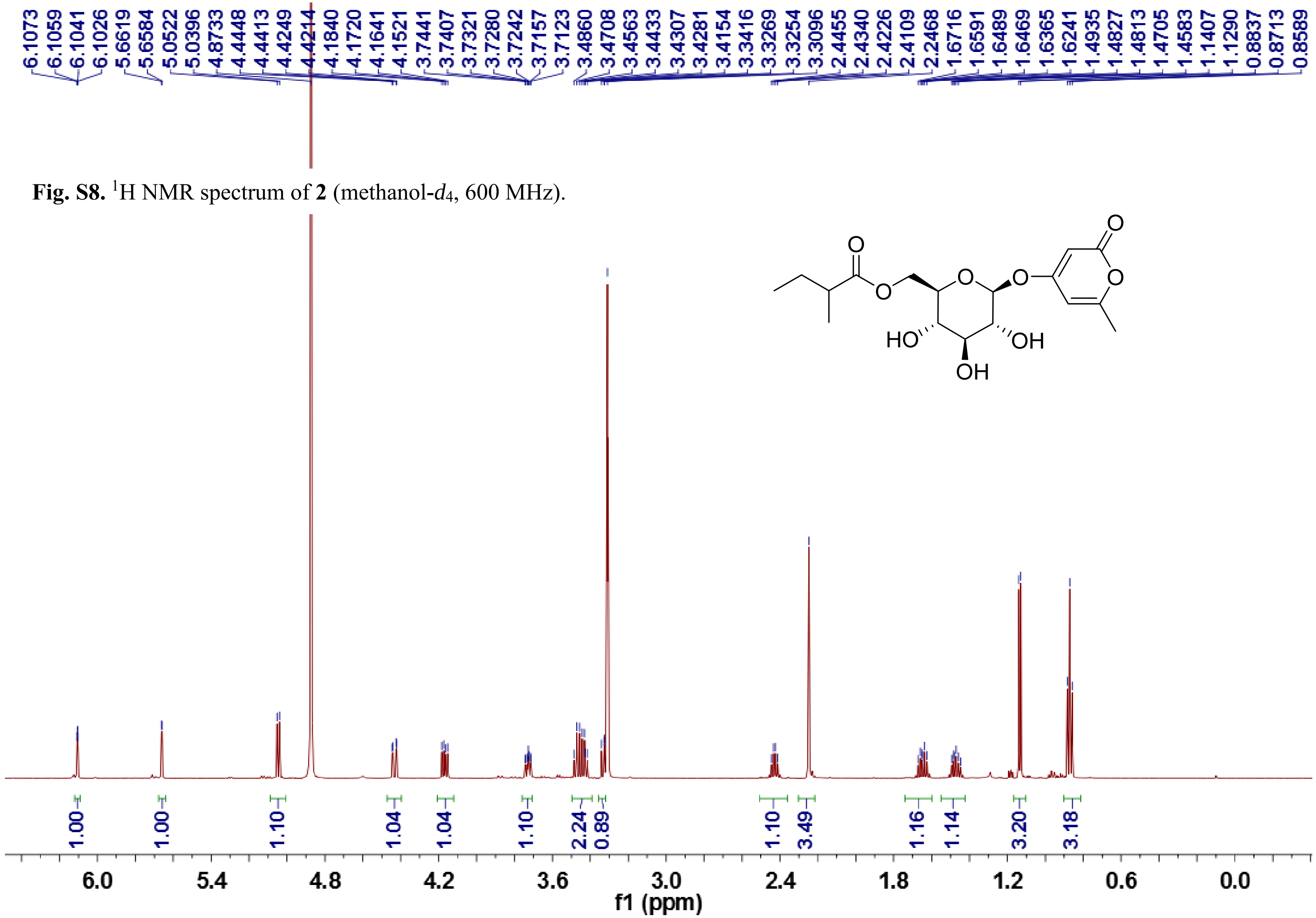

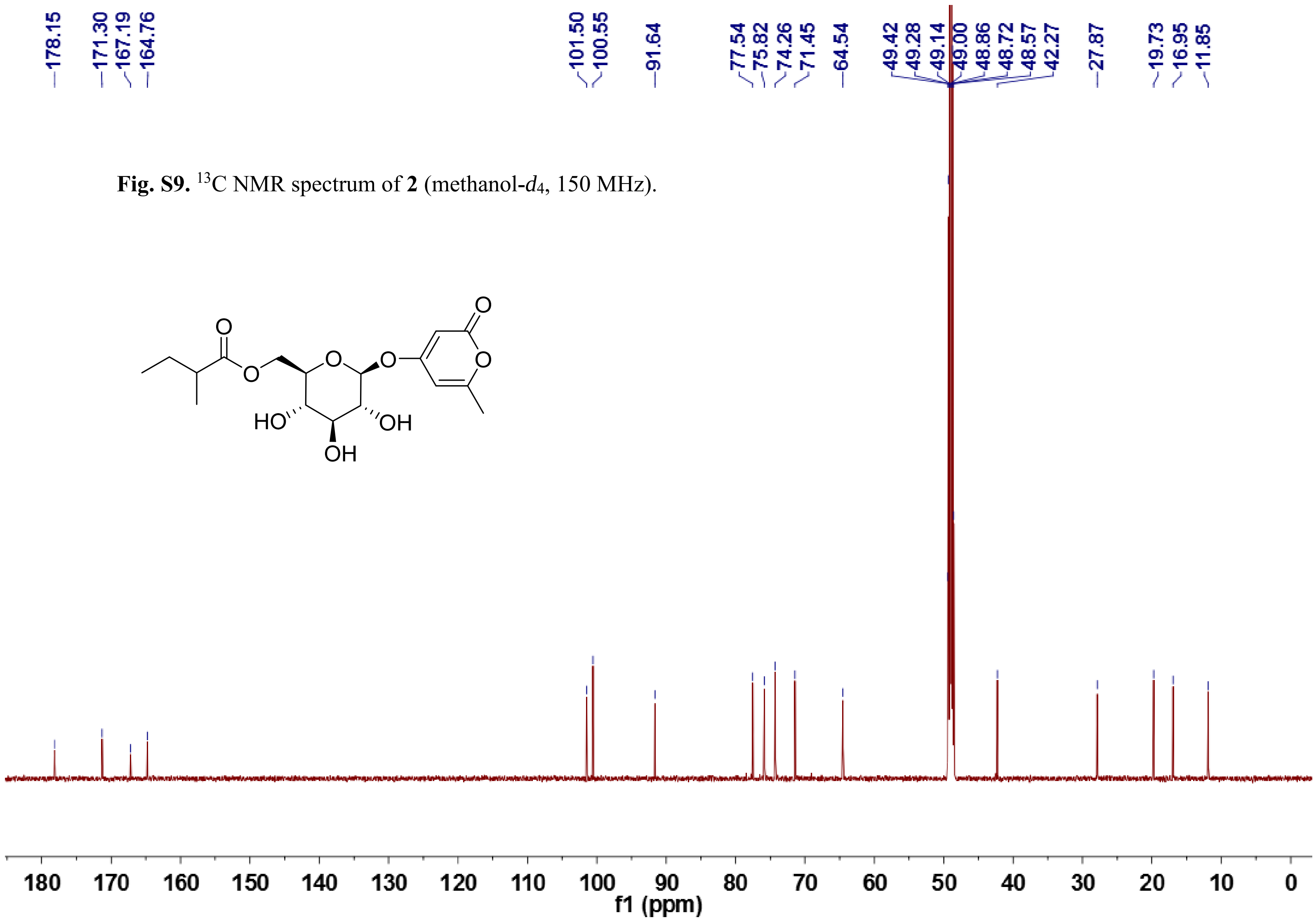

Fig. S9. ^{13}C NMR spectrum of **2** (methanol-d_4, 150 MHz).

Fig. S10. HSQC spectrum of **2**.

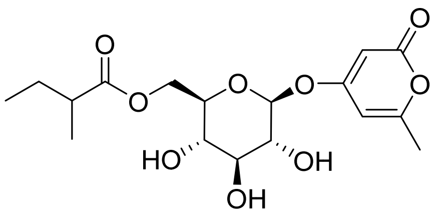

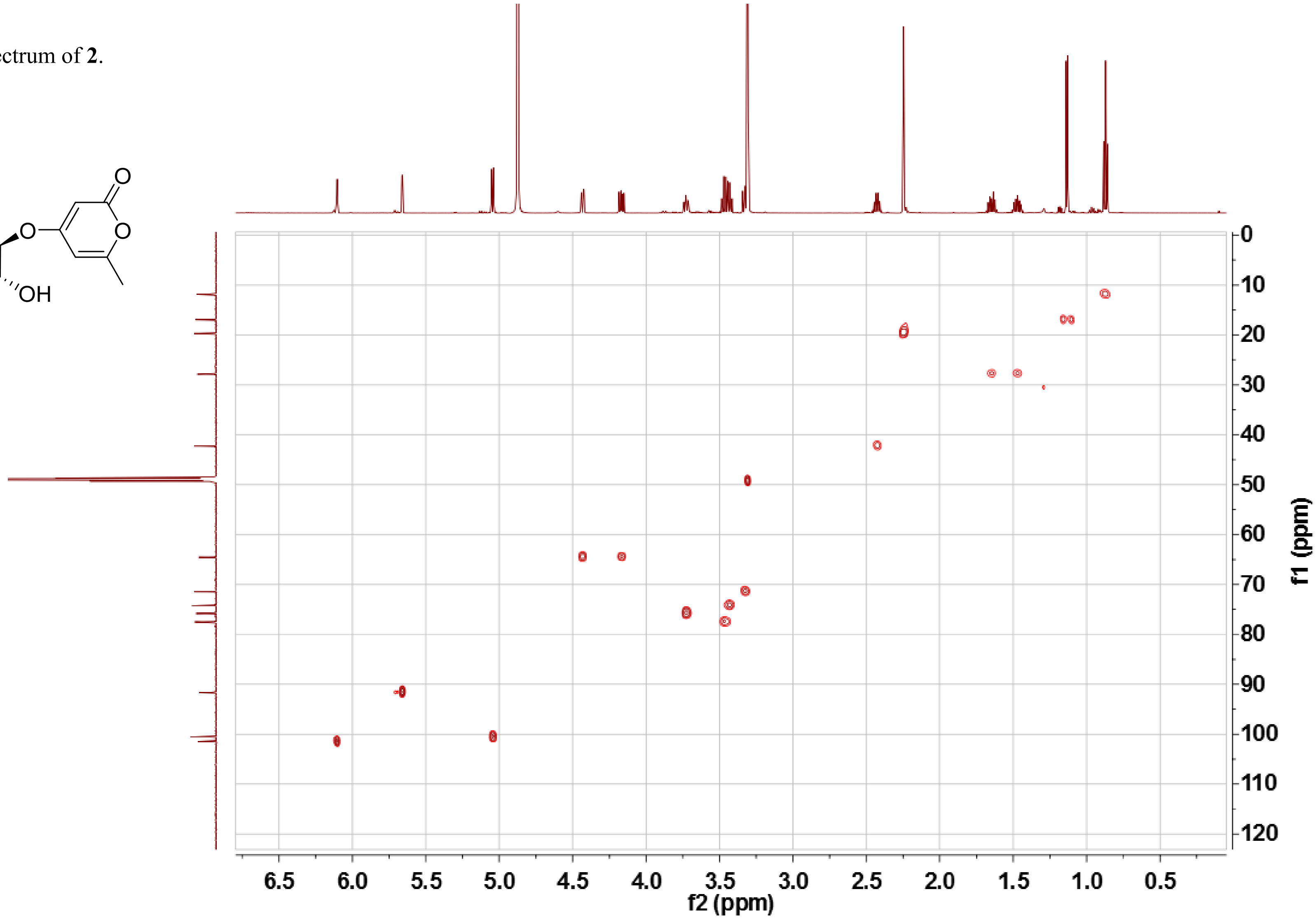

Fig. S11. ^{1}H–^{1}H COSY spectrum of **2**.

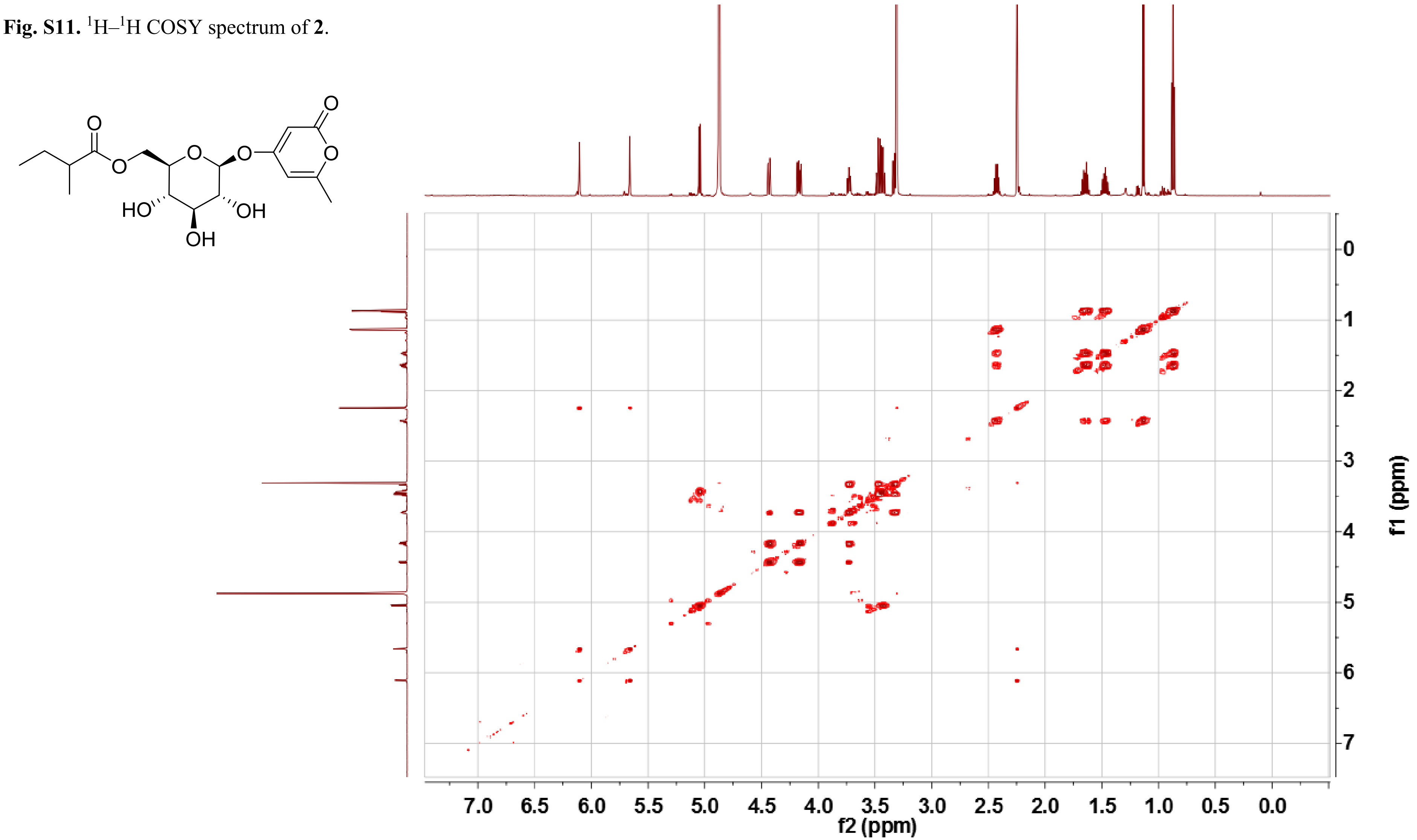

Fig. S12. HMBC spectrum of **2**.

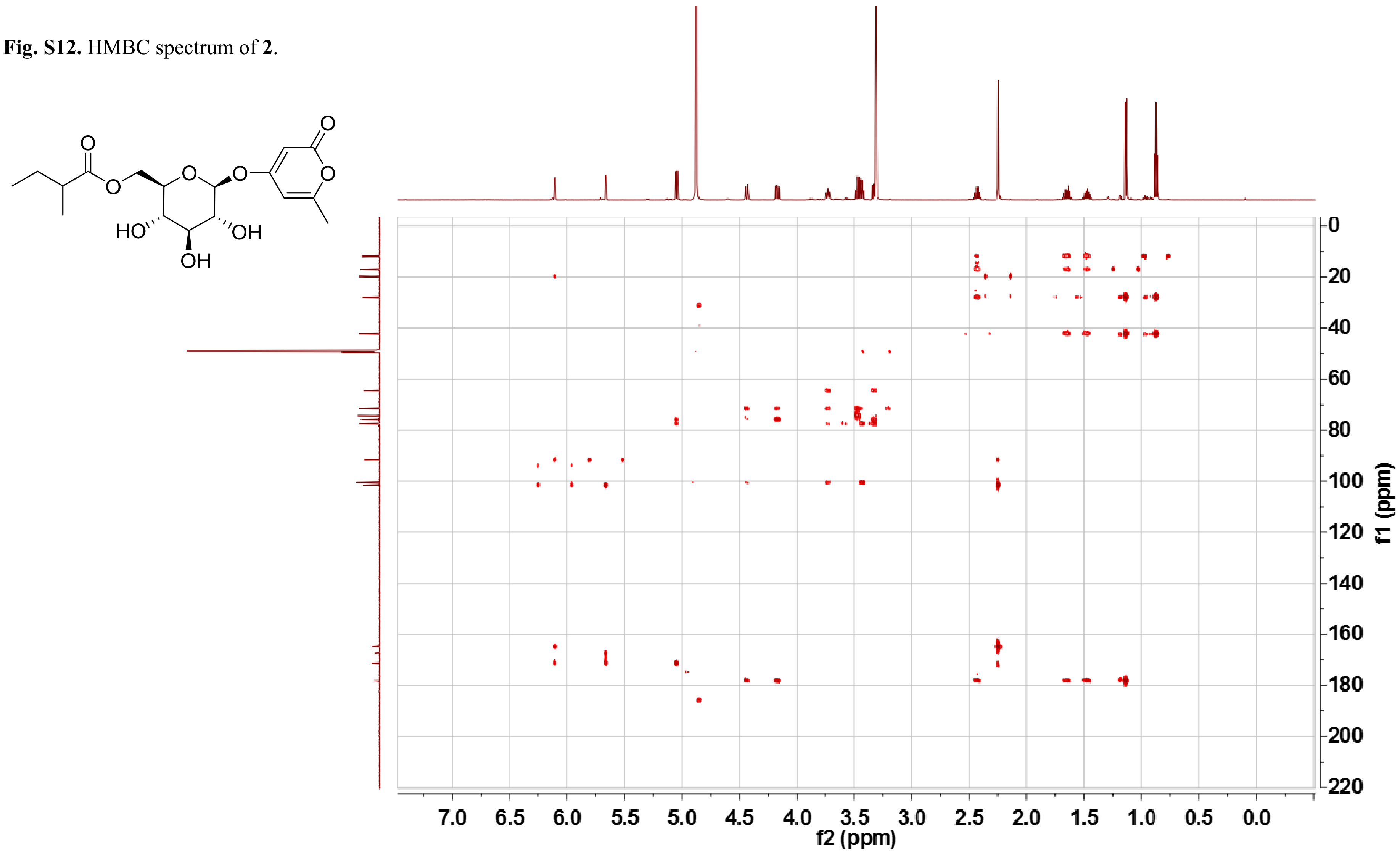

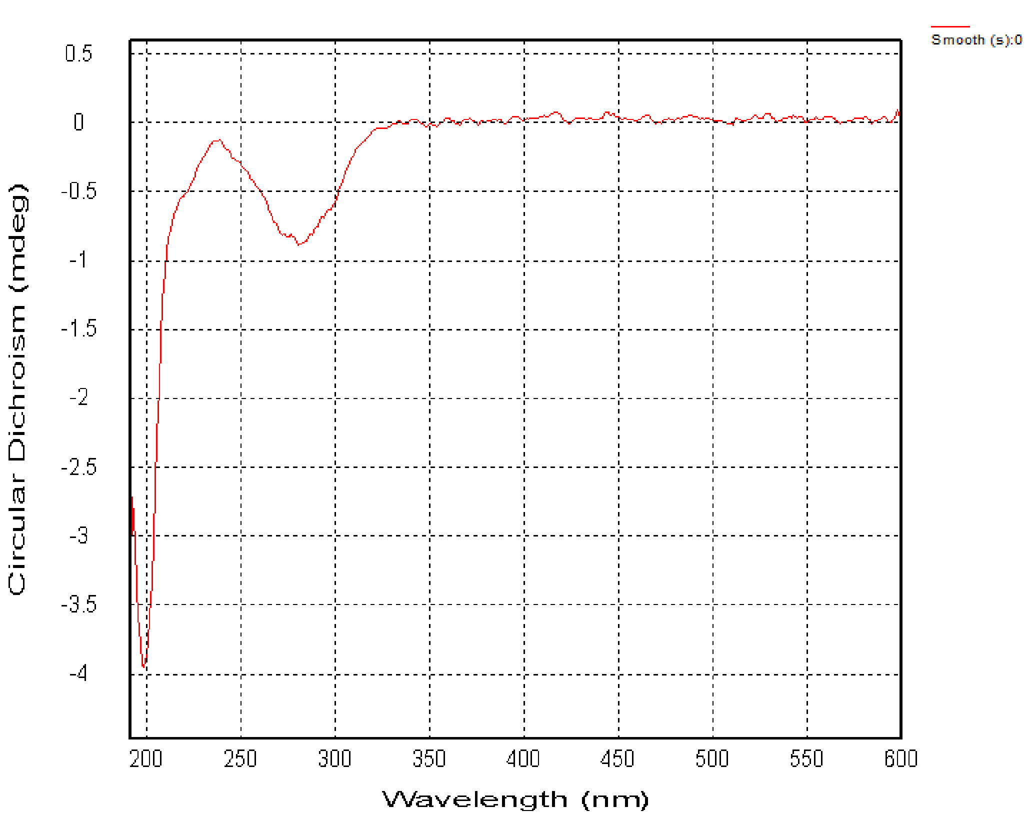

ProBinaryX
Attributes :
- Time Stamp :Wed Jun 05 14:49:31 2019

- File ID : {A75DC47C-174C-4109-B8CB-A19CCABE4B84}
- Is CFR Compliant : false
- Original data has not been modified.
Remarks:
  - User: CD
  - Date: 2019/06/05
  - Instrument: 0547
  - DetectorType: LAAPD
  - DichOS Calibration Correction Curve: 0547/2
  - HV (CDDC channel): 0 v
  - Time per point: 0.25 s
  - Description: pet15
  - Concentration: 0.11 mg/ml CH_3OH
  - Pathlength: 1 mm
  - Temperature: ---- C

Settings:
  - Time-per-point: 0.25s (25us x 10000)
  - SE
  - Wavelength: 192nm - 600nm
  - Step Size: 1nm
  - Bandwidth: 1nm
  - 3 repeats in set.
  - -iter option selected

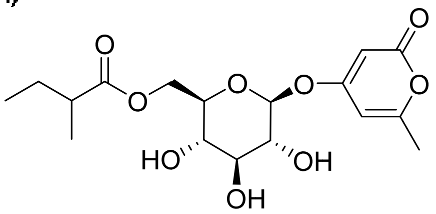

Fig. S13. ECD spectrum of **2.**

**Data Filename**	20200115ESIA5.d	**Sample Name**	pet15
**Sample Type**	Sample	**Position**	
**Instrument Name**	Agilent G6230 TOF MS	**User Name**	KIB
**Acq Method**	ESI.m	**Acquired Time**	1/15/2020 10:40:52 AM
**IRM Calibration Status**	Success	**DA Method**	ESI.m
**Comment**			

**Sample Group**		**Info.**
**Acquisition SW Version**	6200 series TOF/6500 series Q-TOF B.05.01 (B5125.2)	

User Spectra

Fragmentor Voltage	Collision Energy	Ionization Mode
200	0	ESI

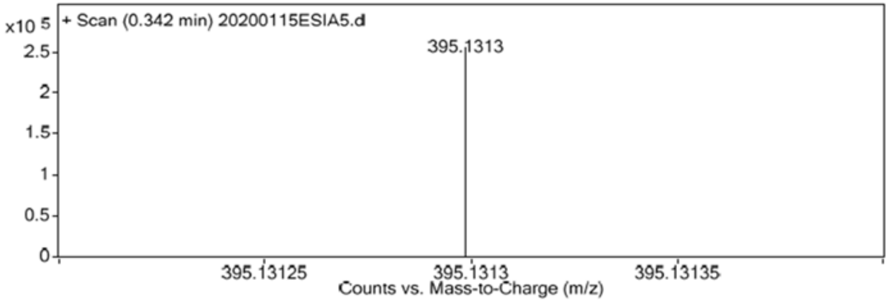

Peak List

m/z	z	Abund	Formula	Ion
121.0509	1	225586.13		
274.2735		25251.2		
395.1313	1	255416.91	C17 H24 Na O9	M+
396.1342	1	47818.17	C17 H24 Na O9	M+
411.105	1	56087.74		
767.2726	1	147883.42		
768.2761	1	51498.23		
783.246	1	23944.63		
922.0098	1	240057.48		
923.0123	1	44657.02		

Formula Calculator Element Limits

Element	Min	Max
C	0	200
H	0	400
O	0	13
Na	1	1

Formula Calculator Results

Formula	CalculatedMass	Mz	Diff.(mDa)	Diff. (ppm)	DBE
C17 H24 Na O9	395.1318	395.1313	0.5	1.3	5.5

--- End Of Report ---

Fig. S14. HRESIMS spectrum of **2.**

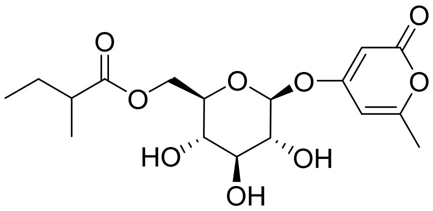

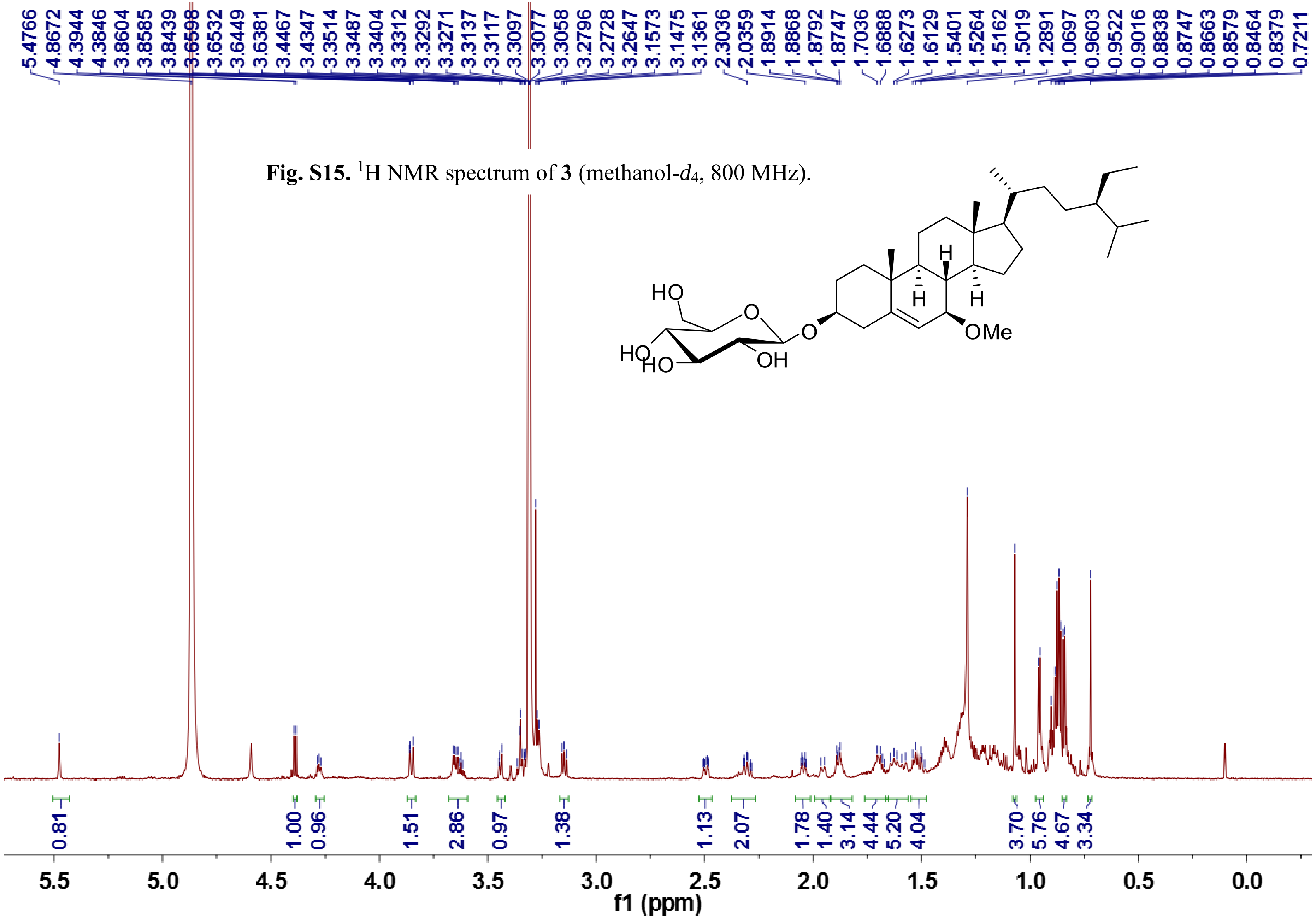

Fig. S15. ^{1}H NMR spectrum of **3** (methanol-d_4, 800 MHz).

Fig. S16. ^{13}C NMR spectrum of **3** (methanol-d_4, 200 MHz).

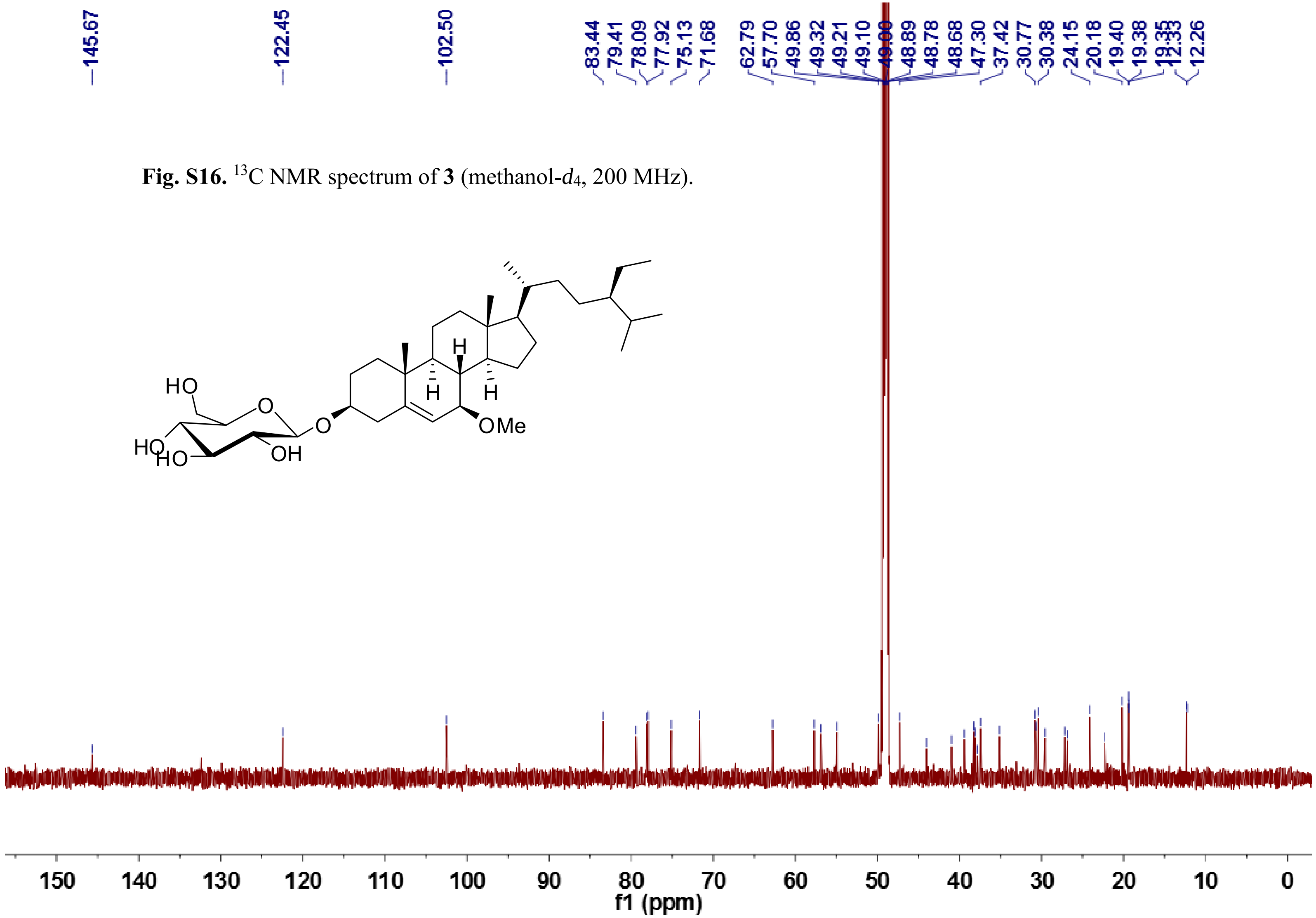

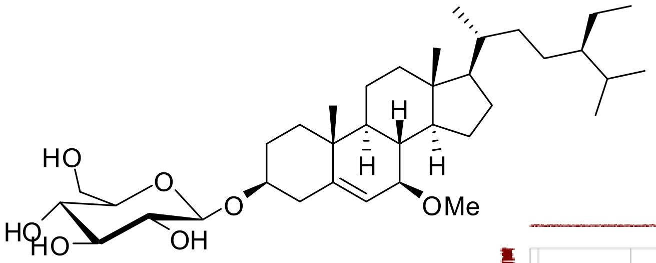

Fig. S17. HSQC spectrum of **3**.

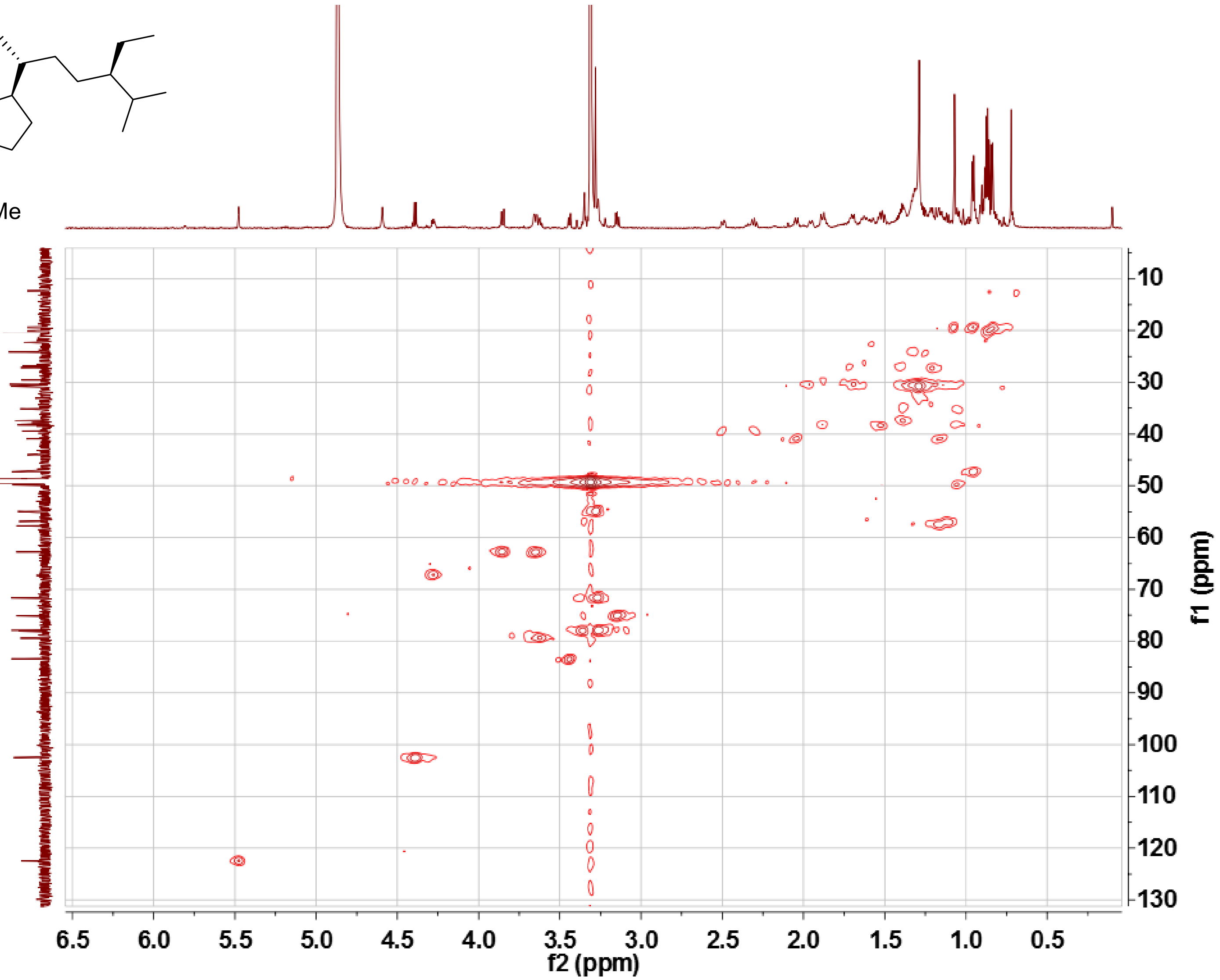

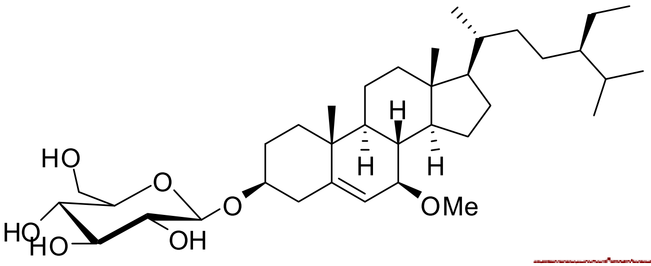

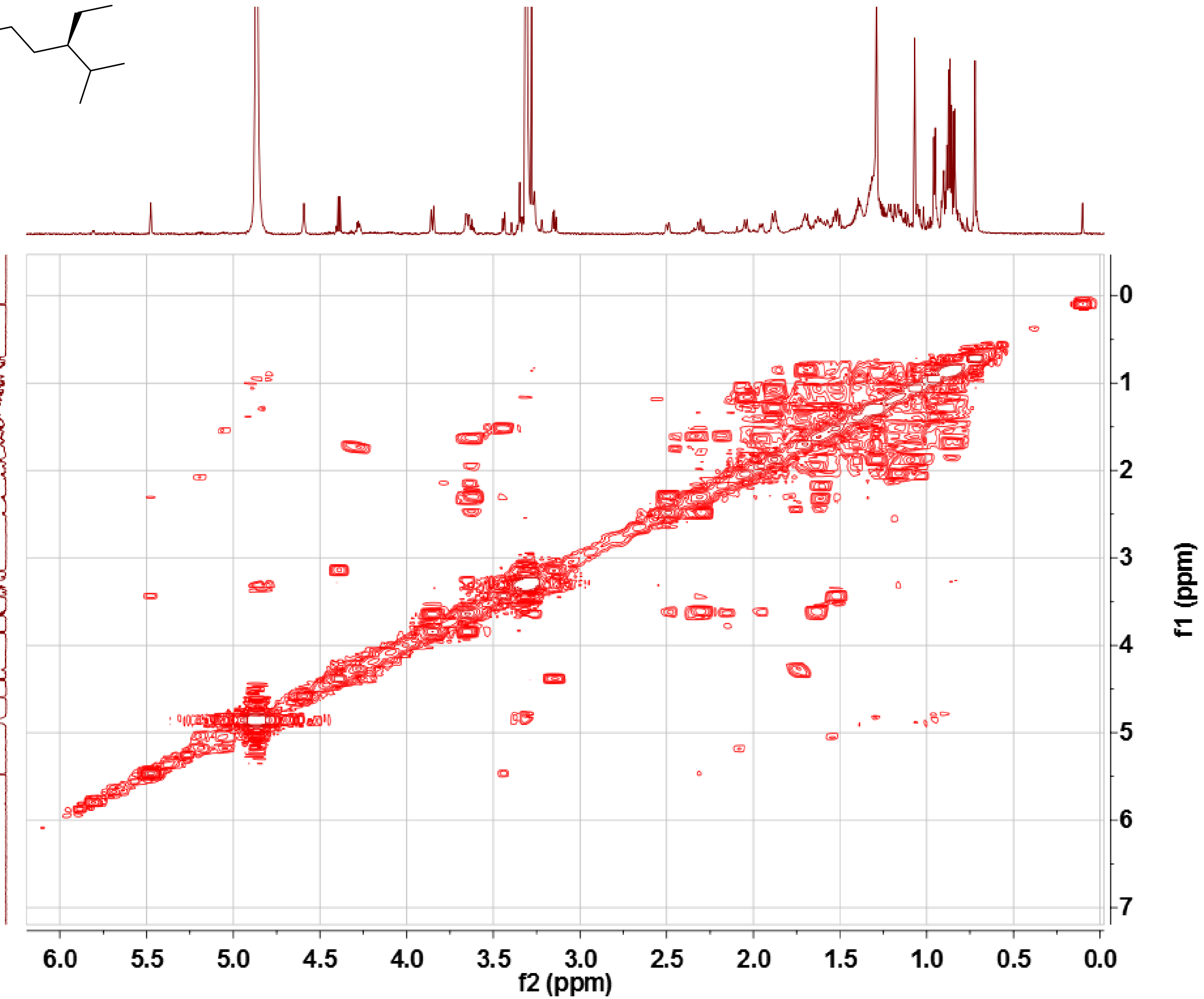

Fig. S18. ^1H–^1H COSY spectrum of **3**.

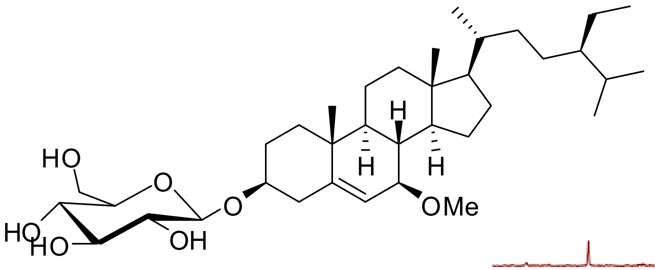

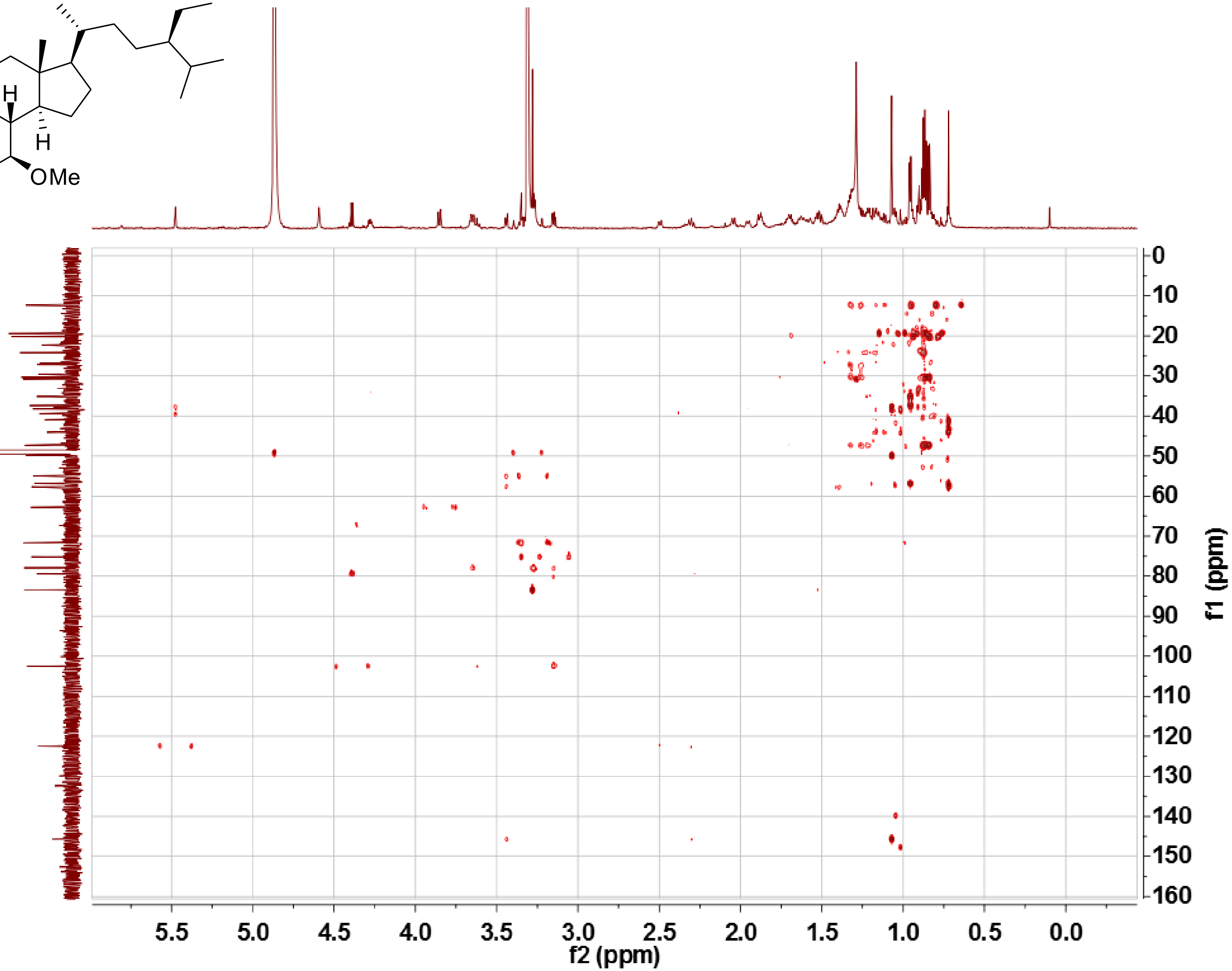

Fig. S19. HMBC spectrum of **3**.

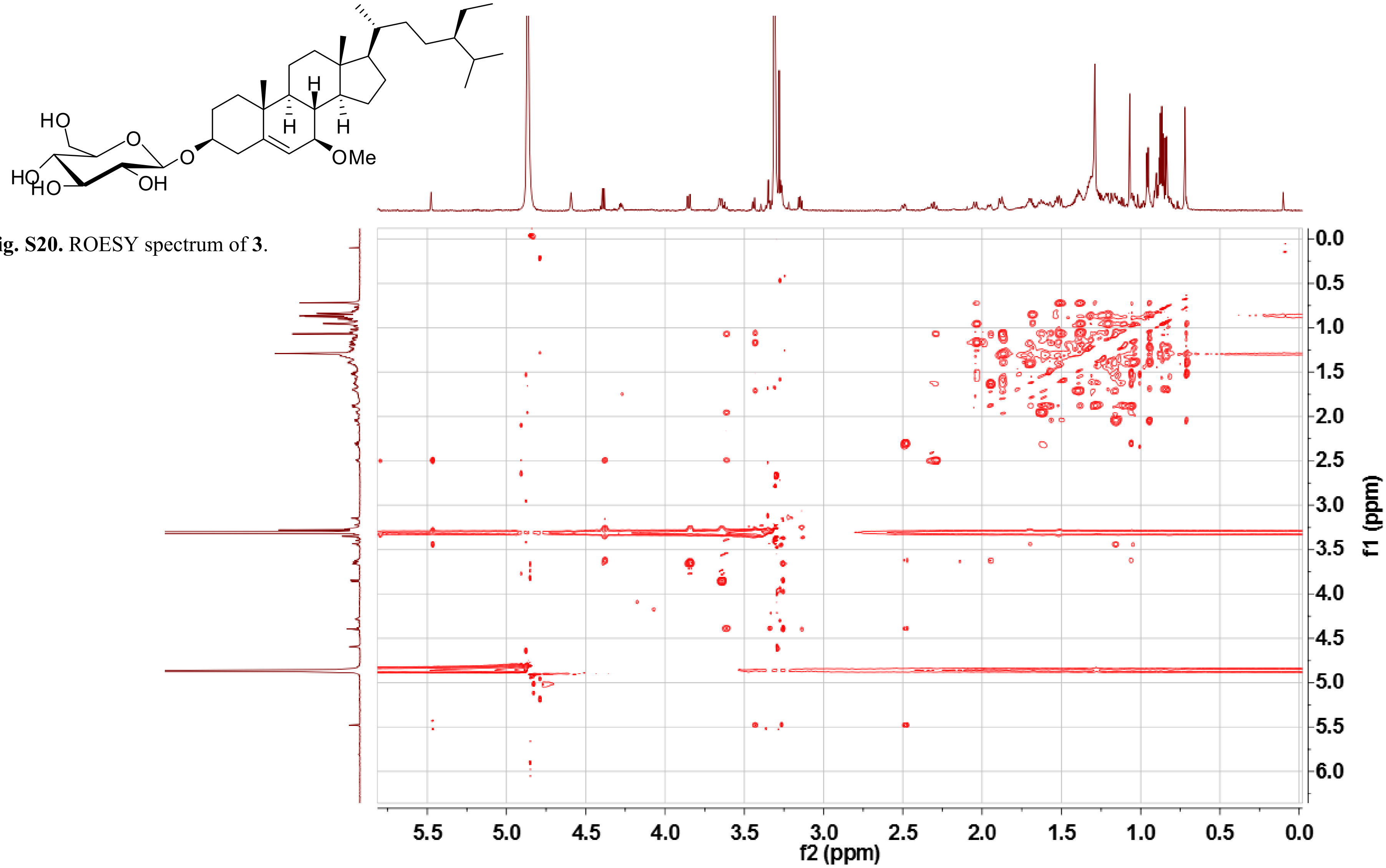

Fig. S20. ROESY spectrum of **3**.

Qualitative Analysis Report

**Data Filename**	190705ESIA1.d	**Sample Name**	pet21
**Sample Type**	Sample	**Position**	
**Instrument Name**	Agilent G6230 TOF MS	**User Name**	KIB
**Acq Method**	ESI.m	**Acquired Time**	7/3/2019 2:17:32 PM
**IRM Calibration Status**	Success	**DA Method**	ESI.m
**Comment**			

Sample Group **Info.**

Acquisition SW Version 6200 series TOF/6500 series Q-TOF B.05.01 (B5125.2)

User Spectra

Fragmentor Voltage	Collision Energy	Ionization Mode
200	0	ESI

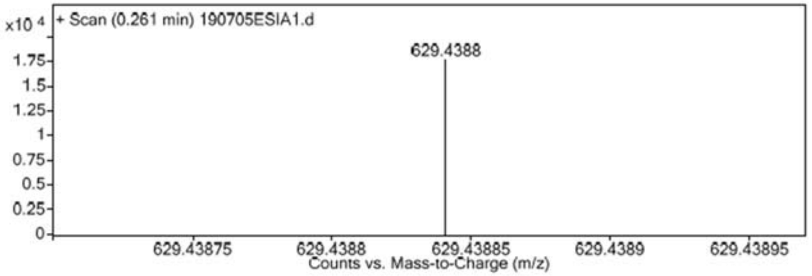

Fig. S21. HRESIMS spectrum of **3.**

Peak List

m/z	z	Abund	Formula	Ion
102.1285	1	79498.65		
105.0425		42448.59		
121.0509	1	61748.42		
205.06		19612.16		
246.0866		23848.63		
441.2974	1	30151.79		
482.3242	1	24195.02		
629.4388	1	17568.13	C36 H62 Na O7	M+
859.6068	1	56858.44		
860.6099	1	33247.41		

Formula Calculator Element Limits

Element	Min	Max
C	0	200
H	0	400
O	3	10
Na	1	1

Formula Calculator Results

Formula	CalculatedMass	Mz	Diff.(mDa)	Diff. (ppm)	DBE
C36 H62 Na O7	629.4393	629.4388	0.5	0.8	5.5

--- End Of Report ---

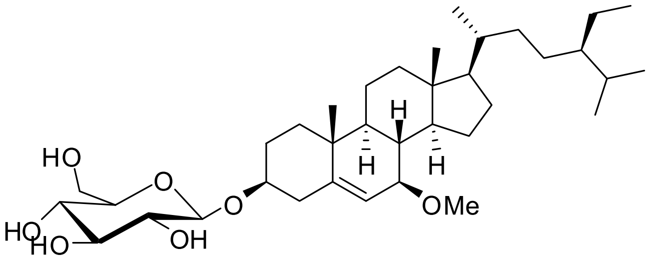

Supplement: Supplementary file 1 — Supplementary file1 (PDF 1402 kb) [file 13659_2020_265_MOESM1_ESM.pdf]
